# The genus *Arthrinium* (*Ascomycota, Sordariomycetes, Apiosporaceae*) from marine habitats from Korea, with eight new species

**DOI:** 10.1186/s43008-021-00065-z

**Published:** 2021-06-01

**Authors:** Sun Lul Kwon, Myung Soo Park, Seokyoon Jang, Young Min Lee, Young Mok Heo, Joo-Hyun Hong, Hanbyul Lee, Yeongseon Jang, Ji-Hyun Park, Changmu Kim, Gyu-Hyeok Kim, Young Woon Lim, Jae-Jin Kim

**Affiliations:** 1 Division of Environmental Science & Ecological Engineering, College of Life Science & Biotechnology, Korea University, Seoul, 02841 South Korea; 2 School of Biological Sciences and Institute of Microbiology, Seoul National University, Seoul, 08826 South Korea; 3 Division of Wood Chemistry and Microbiology, National Institute of Forest Science, Seoul, 02455 South Korea; 4 Microorganism Resources Division, National Institute of Biological Resources, Incheon, 22689 South Korea

**Keywords:** Fungal diversity, Marine fungi, Multigene phylogeny, Eight new taxa

## Abstract

Species of *Arthrinium* are well-known plant pathogens, endophytes, or saprobes found in various terrestrial habitats. Although several species have been isolated from marine environments and their remarkable biological activities have been reported, marine *Arthrinium* species remain poorly understood. In this study, the diversity of this group was evaluated based on material from Korea, using morphological characterization and molecular analyses with the internal transcribed spacer (ITS) region, β-tubulin (TUB), and translation elongation factor 1-alpha (TEF). A total of 41 *Arthrinium* strains were isolated from eight coastal sites which represented 14 species. Eight of these are described as new to science with detailed descriptions.

**Supplementary Information:**

The online version contains supplementary material available at 10.1186/s43008-021-00065-z.

## INTRODUCTION

The genus *Arthrinium*, which belongs to *Apiosporaceae* in *Xylariales* (class *Sordariomycetes* in *Ascomycota*), was first recognized and established more than 200 years ago, with *A. caricicola* as type species (Schmidt and Kunze 1817). To date, it comprises approximately 88 species worldwide (Index Fungorum: http://www.indexfungorum.org).

*Arthrinium* species have traditionally been classified based on morphological characteristics such as conidial shape, conidiophores, and the presence or absence of sterile cells and setae (Schmidt & Kunze 1817; Hughes 1953; Minter 1985). Among these characteristics, conidial shape appears to be diagnostic for distinguishing species (Singh et al. 2013). However, morphological variation is often observed depending on the growth substrate and incubation period (Crous & Groenewald 2013). As such, species identification based on morphological characteristics is problematic and impractical. To address this problem, DNA sequences of the internal transcription spacer (ITS), translation elongation factor 1-alpha (TEF), and β-tubulin gene (TUB) were employed to delimit and recognize closely related *Arthrinium* species and infer their phylogenetic relationships (Crous & Groenewald 2013).

*Arthrinium* species have been globally reported as endophytes, plant pathogens, and saprobes and are commonly isolated from various terrestrial environments, including air, plants, and soil (Kim et al. 2011; Crous & Groenewald 2013; Wang et al. 2018). More recently, isolation from various marine environments, including seawater, seaweed, and the inner tissues of marine sponges, has been reported (Miao et al. 2006; Tsukamoto et al. 2006; Suryanarayanan 2012; Flewelling et al. 2015; Hong et al. 2015; Wei et al. 2016; Elissawy et al. 2017; Li et al. 2017). *Arthrinium* species isolated from sponges, egg masses of sailfin sandfish, and seaweeds showed promising bioactive properties, including high enzymatic activity, antifungal activity, and antioxidant capacity (Elissawy et al. 2017; Li et al. 2017; Park et al. 2018). Some species (*A. arundinis, A. phaeospermum, A. rasikravindrae, A. sacchari, and A. saccharicola*) have been detected in both marine and terrestrial environments (Wang et al. 2018). Whether these species have specific adaptations to survive in seawater requires further investigation. A recent study showed that marine *Arthrinium* species developed strategies to adapt to marine environments, such as a symbiotic partnership with seaweed (Heo et al. 2018). In marine systems, dissolved organic matter in seawater can absorb ultraviolet radiation and produce reactive oxygen species (ROS), which cause oxidative stress on marine microorganisms (Mopper & Kieber 2000). Heo et al. (2018) detected relatively high antioxidant activity and radical-scavenging activity in marine-derived *Arthrinium* species. The antifungal activity of seaweed-pathogenic fungi has also been studied (Hong et al. 2015; Heo et al. 2018). *Arthrinium saccharicola* (KUC21342) has the potential to inhibit the growth of *Asteromyces cruciatus,* a pathogenic fungus that attacks brown algae (Heo et al. 2018). The discovery of the promising bioactivities of marine *Arthrinium* species was one of the reasons motivating our subsequent investigation of the diversity of marine *Arthrinium* in Korea.

Six species of *Arthrinium* have previously been reported from marine environments in Korea: *A. arundinis, A. marii, A. phaeospermum, A. rasikravindrae, A. sacchari,* and *A. saccharicola* (Hong et al. 2015; Heo et al. 2018; Park et al. 2018). However, many marine species remain unidentified owing to the lack of resolution in ITS-based phylogenies and the paucity of morphological characteristics. The aim of this study was to investigate marine *Arthrinium* species from coastal environments in Korea and to identify them using morphological characteristics and multigene phylogenies (ITS, TEF, and TUB).

## MATERIALS AND METHODS

### Sampling and isolation

The seaweed *Sargassum fulvellum* and unidentified seaweeds were collected from two locations, Taean-gun on the west coast of Korea and Jeju Island south of Korea. To isolate the fungi, the seaweeds were washed with distilled water and cut into small pieces (approximately 5 mm diam) using a sterile surgical blade. The pieces were treated with 70% ethanol for 60 s and washed in sterile distilled water for 10 s. Each piece was placed on 2% malt extract agar (MEA) supplemented with 0.01% streptomycin and 0.01% ampicillin to inhibit bacterial growth. The plates were incubated at 25 °C for 7–15 d. Suspected *Arthrinium* colonies were transferred onto potato dextrose agar (PDA, Difco, Sparks, MD, USA) plates. The colonies were subsequently identified as belonging to *Arthrinium* based on ITS sequences (see below). A total of 14 *Arthrinium* strains were isolated in this study and an additional 27 *Arthrinium* strains were obtained from the Seoul National University Fungus Collection (SFC), Seoul, Korea. Each strain is stored in 20% glycerol at − 80 °C in the Korea University Fungus Collection (KUC), Seoul, Korea. Type specimens were deposited in the Korean Collection for Type Culture, Daejeon, Korea (KCTC), with ex-type living cultures deposited in KUC.

### DNA extraction, PCR amplification, and sequencing

Genomic DNA was extracted using an Accuprep Genomic DNA extraction kit (Bioneer, Korea) according to the manufacturer’s protocol. PCR targeting the ITS, TUB, and TEF regions was carried out according to a previously described method (Hong et al. 2015). For the ITS region, the primers ITS1F and ITS4/LR3 were used (White et al. 1990; Gardes & Bruns 1993); for TUB, we employed Bt2a/T10 and Bt2b/T2 (Glass & Donaldson 1995; O’Donnell & Cigelnik 1997), and for TEF, we used EF1-728F and EF2 (O’Donnell et al. 1998; Carbone & Kohn 1999). All PCR products were checked on a 1% agarose gel and purified with the AccuPrep PCR/Gel DNA Purification Kit (Bioneer, Seoul, Korea). DNA sequencing was performed at Macrogen (Seoul, Korea) on an ABI3730 automated DNA Sequencer (Applied Biosystems, Foster City, CA) using the same set of primers for each locus. Additional DNA sequences of some strains were obtained from previous studies (Hong et al. 2015; Heo et al. 2018). All new sequences generated in this study were deposited in GenBank (Table 1).

**Table 1:** A list of all the strains included in the phylogenetic analysis

Identity	Culture no.^a^	Isolation source	Location	GenBank accession no.^b^		
				ITS	TUB	TEF
***A. agari*** **sp. nov.**	KUC21333^T^ = SFC20161014-M18	*Agarum cribrosum*	Yangyang-gun, Korea	MH498520	MH498478	MH544663
	KUC21361	*Agarum cribrosum*	Yangyang-gun, Korea	**MH498519**	**MH498477**	**MN868914**
	KUC21362	*Agarum cribrosum*	Yangyang-gun, Korea	**MH498518**	**MH498476**	**MN868915**
	KUC21363	*Agarum cribrosum*	Yangyang-gun, Korea	**MH498517**	**MH498475**	**MN868916**
	KUC21364	*Agarum cribrosum*	Yangyang-gun, Korea	**MH498516**	**MH498474**	**MN868917**
***A. arctoscopi*** **sp. nov.**	KUC21331^T^ = SFC20200506-M05	Egg of *Arctoscopus japonicus*	Goseong-gun, Korea	**MH498529**	**MH498487**	**MN868918**
	KUC21344	Egg of *Arctoscopus japonicus*	Goseong-gun, Korea	**MH498528**	**MH498486**	**MN868919**
	KUC21345	Egg of *Arctoscopus japonicus*	Goseong-gun, Korea	**MH498527**	**MH498485**	**MN868920**
	KUC21346	Egg of *Arctoscopus japonicus*	Goseong-gun, Korea	**MH498526**	**MH498484**	**MN868921**
	KUC21347	Egg of *Arctoscopus japonicus*	Goseong-gun, Korea	**MH498525**	**MH498483**	**MN868922**
*A. arundinis*	CBS 124788	Living leaves of *Fagus sylvatica*	Basel, Switzerland	KF144885	KF144975	KF145017
	CBS 114316	Leaf of *Hordeum vulgare*	Shabestar, Iran	KF144884	KF144974	KF145016
	KUC21261	*Sargassum fulvellum*	Jeju-do, Korea	KT207779	MH498511	MH544683
	KUC21229	*Sargassum fulvellum*	Jeju-do, Korea	KT207747	MH498512	MH544684
	KUC21337	Beach Sand	Muan-gun, Korea	MH498551	MH498509	MH544682
*A. aureum*	CBS 244.83	Air	Barcelona, Spain	AB220251	KF144981	KF145023
*A. balearicum*	AP24118^T^ = CBS 145129	Undetermined *Poaceae*	Liucmajor, Spain	MK014869	MK017975	–
*A. bambusae*	LC7106	Leaf of bamboo	China	KY494718	KY705186	KY806204
	LC7107	Leaf of bamboo	China	KY494719	KY705187	KY705117
*A. camelliae-sinensis*	LC5007	*Camellia sinensis*	China	KY494704	KY705173	KY705103
	LC8181	*Brassica capestris*	China	KY494761	KY705229	KY705157
*A. descalsii*	AP3118A^T^ = CBS 145130	*Ampelodesmos mauritanicus*	Spain	MK014870	MK017976	–
*A. dichotomanthi*	LC4950	*Dichotomanthus tristaniaecarpa*	China	KY494697	KY705167	KY705096
	LC8175	*Dichotomanthus tristaniaecarpa*	China	KY494755	KY705223	KY705151
*A. esporlense*	AP16717 ^T^ = CBS 145136	*Phyllostachys aurea*	Spain	MK014878	MK017983	–
*A. euphorbiae*	IMI 285638b	*Bambusa* sp.	Bangladesh	AB220241	AB220288	–
***A. fermenti*** **sp. nov.**	KUC21289 ^T^	Seaweed	Haenam-gun, Korea	MF615226	MF615231	MH544667
	KUC21288 = SFC20140423-M86	Seaweed	Haenam-gun, Korea	**MF615230**	**MF615235**	**MH544668**
*A. gaoyouense*	CFCC 52301	*Phragmites australis*	China	MH197124	MH236789	MH236793
	CFCC 52302	*Phragmites australis*	China	MH197125	MH236790	MH236794
*A. garethjonesii*	JHB004 = HKAS:96289	Culms of dead bamboo	China	KY356086	–	–
*A. guizhouense*	LC5318	Air in karst cave	China	KY494708	KY705177	KY705107
	LC5322	Air in karst cave	China	KY494709	KY705178	KY705108
*A. gutiae*	CBS 135835	Gut of a grasshopper	India	KR011352	KR011350	KR011351
*A. hispanicum*	IMI 326877	Maritime sand	Spain	AB220242	AB220289	–
*A. hydei*	CBS 114990	Culms of *Bambusa tuldoides*	Tai Po Kau, Hong Kong	KF144890	KF144982	KF145024
	JHB0012 = HKAS:96355	Dead culms of bamboo	China: Kunming	KY356087	–	–
	LC7103	Leaf of bamboo	China	KY494715	KY705183	KY705114
	LC7105	Leaf of bamboo	China	KY494717	KY705185	KY705116
*A. hyphopodii*	MFLUCC 15–0003	Culms of *Bambusa tuldoides*	Thailand	KR069110	–	–
	JHB003 = HKAS:96288	Culms of Bamboo	China: Kunming	KY356088	–	–
*A. hysterinum*	CBS 145133	*Phyllostachys aurea*	Spain	MK014875	MK017981	–
	CBS 145135	*Phyllostachys aurea*	Spain	MK014877	MK017982	–
*A. ibericum*	AP10118 ^T^ = CBS 145137	*Arundo donax*	Portugal	MK014879	MK017984	–
*A. italicum*	AP221017 ^T^ = CBS 145138	*Arundo donax*	Italy	MK014880	MK017985	MK017956
	AP29118 = CBS 145139	*Phragmites australis*	Spain	MK014881	MK017986	–
*A. jiangxiense*	LC2831	Leaf of bamboo	China	KY494686	KY806201	KY705085
	LC4494	*Phyllostachys* sp.	China	KY494690	KY705160	KY705089
*A. kogelbergense*	CBS 113332	Culms of *Cannomois virgata*	Republic of South Africa	KF144891	KF144983	KF145025
	CBS 113333	Dead culms of Restionaceae	Republic of South Africa	KF144892	KF144984	KF145026
***A. koreanum*** **sp. nov.**	KUC21332 ^T^ = SFC20200506-M06	Egg of *Arctoscopus japonicus*	Goseong-gun, Korea	MH498524	MH498482	MH544664
	KUC21348	Egg of *Arctoscopus japonicus*	Goseong-gun, Korea	**MH498523**	**MH498481**	**MN868927**
	KUC21349	Egg of *Arctoscopus japonicus*	Goseong-gun, Korea	**MH498522**	**MH498480**	**MN868928**
	KUC21350	Egg of *Arctoscopus japonicus*	Goseong-gun, Korea	**MH498521**	**MH498479**	**MN868929**
*A. longistromum*	MFLUCC 11–0481	Culms of Decaying bamboo	Thailand	KU940141	–	–
	MFLUCC 11–0479	Culms of Decaying bamboo	Thailand	KU940142	–	–
*A. malaysianum*	CBS 251.29	Stem base of *Cinnamomum camphora*	Malaysia	KF144897	KF144989	KF145031
	CBS 102053	*Macaranga hullettii* stem colonized by ants	Gombak, Malaysia	KF144896	KF144988	KF145030
*A. marii*	KUC21338 = SFC20140423-M01	Seaweed	Muan-gun, Korea	MH498549	MH498507	MH544681
	CBS 113535	Oats	Sweden	KF144898	KF144990	KF145032
	CBS 114803	Culm of *Arundinaria hindsi*	Lung Fu Shan, Hong Kong	KF144899	KF144991	KF145033
***A. marinum*** **sp. nov.**	KUC21328^T^ = SFC20140423-M02	Seaweed	Suncheon-si, Korea	MH498538	MH498496	MH544669
	KUC21353	Seaweed	Suncheon-si, Korea	**MH498537**	**MH498495**	**MN868923**
	KUC21354	Seaweed	Suncheon-si, Korea	**MH498536**	**MH498494**	**MN868924**
	KUC21355	Seaweed	Suncheon-si, Korea	**MH498535**	**MH498493**	**MN868925**
	KUC21356	Seaweed	Suncheon-si, Korea	**MH498534**	**MH498492**	**MN868926**
*A. mediterranei*	IMI 326875	Air	Spain	AB220243	AB220290	–
*A. mytilomorphum*	DAOM 214595	Dead blades of *Andropogon* sp.	India	KY494685	–	–
*A. obovatum*	LC4940	*Lithocarpus* sp.	China	KY494696	KY705166	KY705095
	LC8177	*Lithocarpus* sp.	China	KY494757	KY705225	KY705153
*A. ovatum*	CBS 115042	*Arundinaria hindsii*	Hong Kong	KF144903	KF144995	KF145037
*A. phaeospermum*	KUC21339	*Phragmites australis*	Boseong-gun, Korea	MH498550	MH498508	–
	CBS 114314	Leaf of *Hordeum vulgare*	Marand, Iran	KF144904	KF144996	KF145038
	CBS 114315	Leaf of *Hordeum vulgare*	Shabestar, Iran:	KF144905	KF144997	KF145039
*A. phragmitis*	CPC 18900	Culms of *Phragmites australis*	Bomarzo, Italy	KF144909	KF145001	KF145043
*A. piptatheri*	AP4817A^T^ = CBS 145149	*Piptatherum miliaceum*	Spain	MK014893	–	
	KUC21220	*Sargassum fulvellum*	Jeju-do, Korea	KT207736	KT207636	**MH544672**
	KUC21279	*Sargassum fulvellum*	Jeju-do, Korea	MF615229	MF615234	MH544671
*A. pseudoparenchymaticum*	LC7234	Leaf of bamboo	China	KY494743	KY705211	KY705139
	LC8173	Leaf of bamboo	China	KY494753	KY705221	KY705149
*A. pseudosinense*	CPC 21546	Leaf of bamboo	Utrecht, Netherlands	KF144910	**MN868936**	KF145044
*A. pseudospegazzinii*	CBS 102052	*Macaranga hullettii* stem colonized by ants	Gombak, Malaysia	KF144911	KF145002	KF145045
*A. pterospermum*	CPC 20193	*Lepidosperma gladiatum*	Adelaide, Australia	KF144913	KF145004	KF145046
	CBS 123185	*Machaerina sinclairii*	Auckland, New Zealand	KF144912	KF145003	–
***A. pusillispermum*** **sp. nov.**	KUC21321 ^T^	Seaweed	Taean-gun, Korea	MH498533	MH498491	**MN868930**
	KUC21357	Seaweed	Taean-gun, Korea	**MH498532**	**MH498490**	**MN868931**
*A. qinlingense*	CFCC 52303	*Fargesia qinlingensis*	China	MH197120	MH236791	MH236795
	CFCC 52303	*Fargesia qinlingensis*	China	MH197121	MH236792	MH236796
*A. rasikravindrae*	CBS 337.61	*Cissus* sp.	Netherlands	KF144914	–	–
	CPC 21602	Rice	Thailand	KF144915	–	–
	LC5449	Soil in karst cave	China	KY494713	KY705182	KY705112
	LC7115	Leaf of bamboo	China	KY494721	KY705189	KY705118
	NFCCI2144	Soil	Svalbard	JF326454	–	–
	KUC21327	Egg of *Arctoscopus japonicus*	Goseong-gun, Korea	MH498541	MH498499	MH544670
	KUC21351	Egg of *Arctoscopus japonicus*	Goseong-gun, Korea	**MH498540**	**MH498498**	**MN868932**
*A. sacchari*	KUC21340 = SFC20200506-M04	Egg of *Arctoscopus japonicus*	Goseong-gun, Korea	MH498548	MH498506	MH544680
	CBS 301.49	Bamboo	Indonesia	KF144917	KF145006	KF145048
	CBS 212.30	*Phragmites australis*	Cambridge, United Kingdom	KF144916	KF145005	KF145047
	CBS 372.67	Air	–	KF144918	KF145007	KF145049
*A. saccharicola*	KUC21221	*Sargassum fulvellum*	Hyeopjae Beach, Jeju-do	KT207737	KT207637	MH544679
	KUC21342 = SFC20160407-M06	Egg of *Arctoscopus japonicus*	Goseong-gun, Korea	MH498546	MH498504	**MN868933**
	KUC21343 = SFC20161110-M12	Egg of *Arctoscopus japonicus*	Yeongok-myeon, Gangneung-si	MH498545	MH498503	MH544678
	CBS 191.73	Air	Utrecht, Netherlands	KF144920	KF145009	KF145051
	CBS 463.83	Dead culms of *Phragmites australis*	Harderbos, Netherlands	KF144921	KF145011	KF145053
***A. sargassi*** **sp. nov.**	KUC21228 ^T^	*Sargassum fulvellum*	Jeju-do, Korea	KT207746	KT207644	MH544677
	KUC21232	*Sargassum fulvellum*	Jeju-do, Korea	KT207750	KT207648	MH544676
	KUC21284	*Sargassum fulvellum*	Jeju-do, Korea	MF615228	MF615233	MH544674
	KUC21287	*Sargassum fulvellum*	Jeju-do, Korea	**MF615227**	**MF615232**	**MN868934**
*A. serenense*	IMI 326869	Food, pharmaceutical excipients, atmosphere	Spain	AB220250	AB220297	–
*A. subroseum*	LC7215	Leaf of bamboo	China	KY494740	KY705208	KY705136
	LC7291	Leaf of bamboo	China	KY494751	KY705219	KY705147
***A. taeanense*** **sp. nov.**	KUC21322^T^	Seaweed	Taean-gun, Korea	MH498515	MH498473	MH544662
	KUC21359	Seaweed	Taean-gun, Korea	**MH498513**	**MH498471**	**MN868935**
*A. thailandicum*	MFLUCC 15–0202	Culms of Dead bamboo	Thailand	KU940145	–	–
	LC5630	Rotten wood	China	KY494714	KY806200	KY705113
*A. vietnamense*	IMI 99670	*Citrus sinensis*	Vietnam	KX986096	KY019466	–
*A. xenocordella*	CBS 478.86	Soil	Matopos, Zimbabwe	KF144925	KF145013	KF145055
	LC3486	*Camellia sinensis*	China	KY494687	KY705158	KY705086
*A. yunnanum*	MFLUCC 15–0002	Culms of Decaying bamboo	China	KU940147	–	–
	DDQ00281	*Phyllostachys nigra*	China	KU940148	–	–
*Nigrospora gorlenkoana*	CBS 480.73	*Vitis vinifera*	Kazakhstan	KX986048	KY019456	KY019420

^**T**^ indicates ex-type

^a^ *CBS* Westerdijk Fungal Biodiverity Institute (WI), Utrecht, The Netherlands; *CFCC* China Forestry Culture Collection Centre, Beijing, China; *CPC* Culture collection of Pedro Crous, housed at the Westerdijk Fungal Biodiversity Institute; *DAOM* Canadian Collection of Fungal Cultures, Ottawa, Canada; *HKAS* Herbarium of Cryptogams, Kunming Institute of Botany, Chinese Academy of Sciences, Yunnan, China; *IMI* CABI Bioscience, Eggham, UK; *LC* Personal culture collection of Lei Cai, housed at CAS, China; *MFLUCC* Mae Fah Luang University Culture Collection, Thailand; *NFCCI* National Fungal Culture Collection of India; *DDQ* D.Q. Dai; *JHB* H.B. Jiang; *KUC* the Korea University Fungus Collection, Seoul, Korea; *SFC* the Seoul National University Fungus Collection

^b^ the sequences generated in this study are shown in bold

### Phylogenetic analysis

ITS sequences were assembled, proofread and edited using MEGA v. 7 (Kumar et al. 2016) and subsequently aligned with *Arthrinium* reference sequences from GenBank using MAFFT 7.130 (Katoh and Standley 2013). To adjust the ambiguous alignment manually, maximum likelihood analysis was performed using all sequence where ambiguous regions excluded using G-block. Then, the original sequences were aligned based on the supported clades, and ambiguous regions were manually adjusted.

Maximum likelihood (ML) analyses were conducted using RAxML v. 7.03 (Stamatakis 2006) and a GTR + G model with 1000 bootstrap replicates. Bayesian tree inference (BI) was carried out using MrBayes version 3.2 (Ronquist et al. 2012), with the best model (HKY + I + G) selected for each marker based on the Bayesian information criteria using jModeltest v. 2.1.10 (Darriba et al. 2012). To achieve stationary equilibrium, 20 million trees were generated, and trees were sampled every 1000 generations. The first 25% of the trees was discarded as burn-in, and the remaining 75% was used for calculating posterior probabilities (PP) in the majority rule consensus tree. All analyses were performed on the CIPRES web portal (Miller et al. 2010).

The sequences of the other two loci (TEF and TUB) were individually aligned with *Arthrinium* reference sequences from GenBank using the same approach described for the ITS. ML and BI analyses also followed the above criteria. The models for TEF and TUB were HKY + I + G and K80 + I + G, respectively. The ITS taxa for the multigene tree were different from those of the single ITS tree, so the model test for the ITS region was redone for the multigene analysis. As a result, the SYM + G model was applied to ITS region in the multigene tree. Finally, sequence concatenation was performed using the same methods and models assigned for each locus described above.

### Morphological observation

Strains were grown on oatmeal agar (OA, Difco™), PDA, and MEA at 15, 20, and 25 °C in darkness for 14 d. The culture characteristics, such as surface structure, presence of aerial mycelium and the colour of the mycelium, colour of colony or medium, and sporulation (Crous et al. 2009), were recorded. Colors and the corresponding codes were evaluated according to the Munsell color chart (Munsell Color, 2009). To determine fungal growth rates, the diameter of each colony was measured every 24 h, and each measurement was performed in triplicate. Microscopic characters were observed with an Olympus BX51 light microscope (Olympus, Tokyo, Japan). Samples were mounted in water to take pictures of conidiophores and conidia, and pictures were taken using a DP20 microscope camera (Olympus, Tokyo, Japan). At least 30 individuals were measured for each microscopic character. To illustrate the range of variation, 5% of the extreme measurements from each end of the range are given in parentheses.

Scanning electron microscope (SEM) was used to observe detailed morphological characters. Colonies sporulating abundantly on PDA, MEA, and OA were freeze-dried. Ion coating and observation were performed by Wooyoung Solution Inc. (Suwon, Korea), using an S-5200 scanning electron microscope (Hitachi, Tokyo, Japan). The SEM images were taken under 1500x to 8000x magnifications.

## RESULTS

A total of 41 *Arthrinium* strains were identified, representing six known and eight new species. Of these strains, 26 were isolated from various seaweeds, 14 from the eggs of sailfin sandfish, and one from beach sand. The dominant species were three of the new species, *A. agari* (5 strains), *A. arctoscopi* (5 strains), and *A. marinum* (5 strains) (Table 1).

**Fig. 1:**
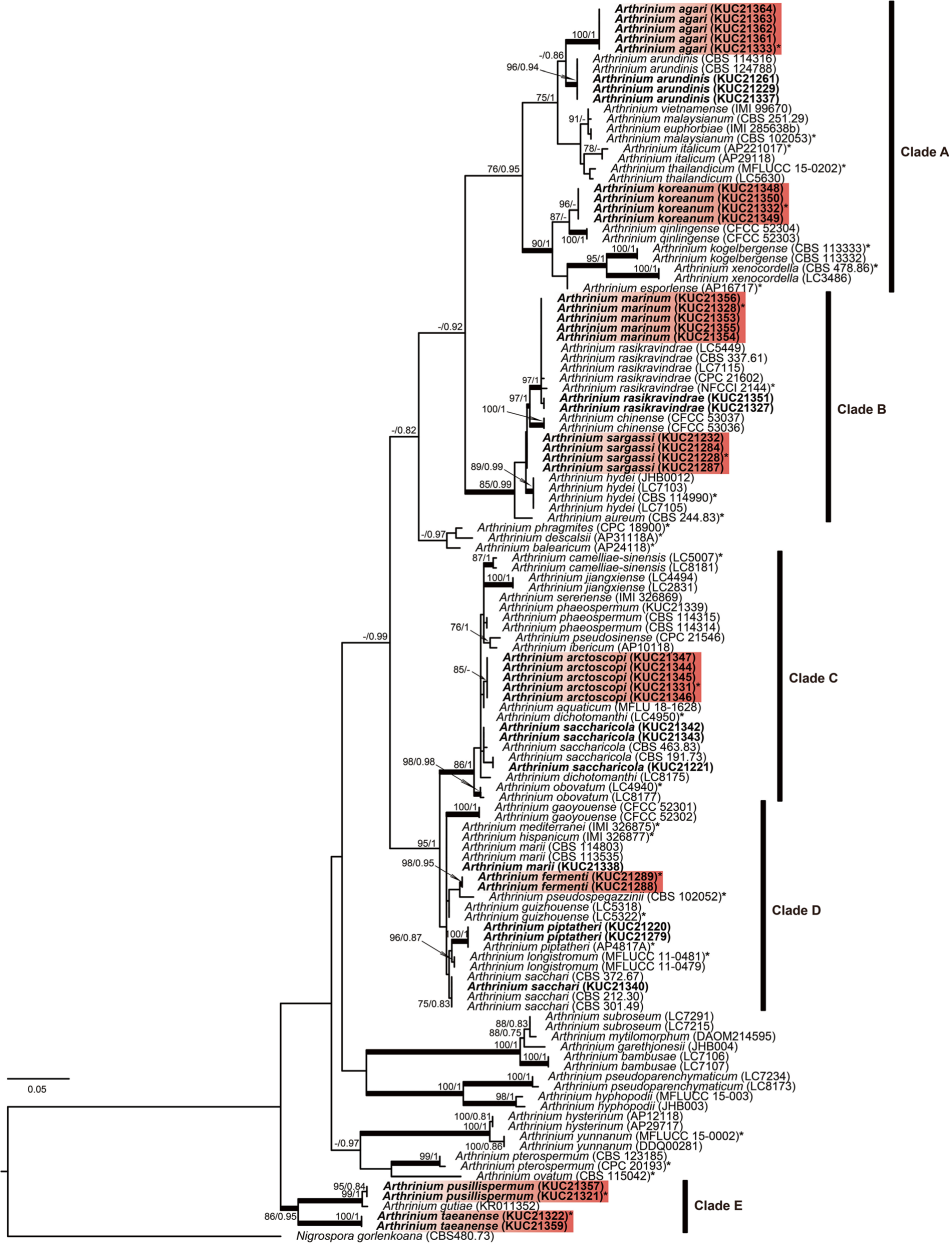
ML tree based on the ITS region. The numbers at the nodes indicate ML bootstrap support (BS) > 75% and Bayesian posterior probabilities (PP) > 0.75 as BS/PP. The thickened branches indicate support greater than 85% for BS and 0.95 for PP. A hyphen (‘-‘) indicates values of BS < 70% or PP < 0.75. Ex-holotype strains are indicated with asterisks (‘*’). The fungal cultures examined in this study are shown in bold. Red boxes indicate the novel species. The numbers in the brackets indicate strain number. The scale bar indicates the nucleotide substitutions per position

**Fig. 2:**
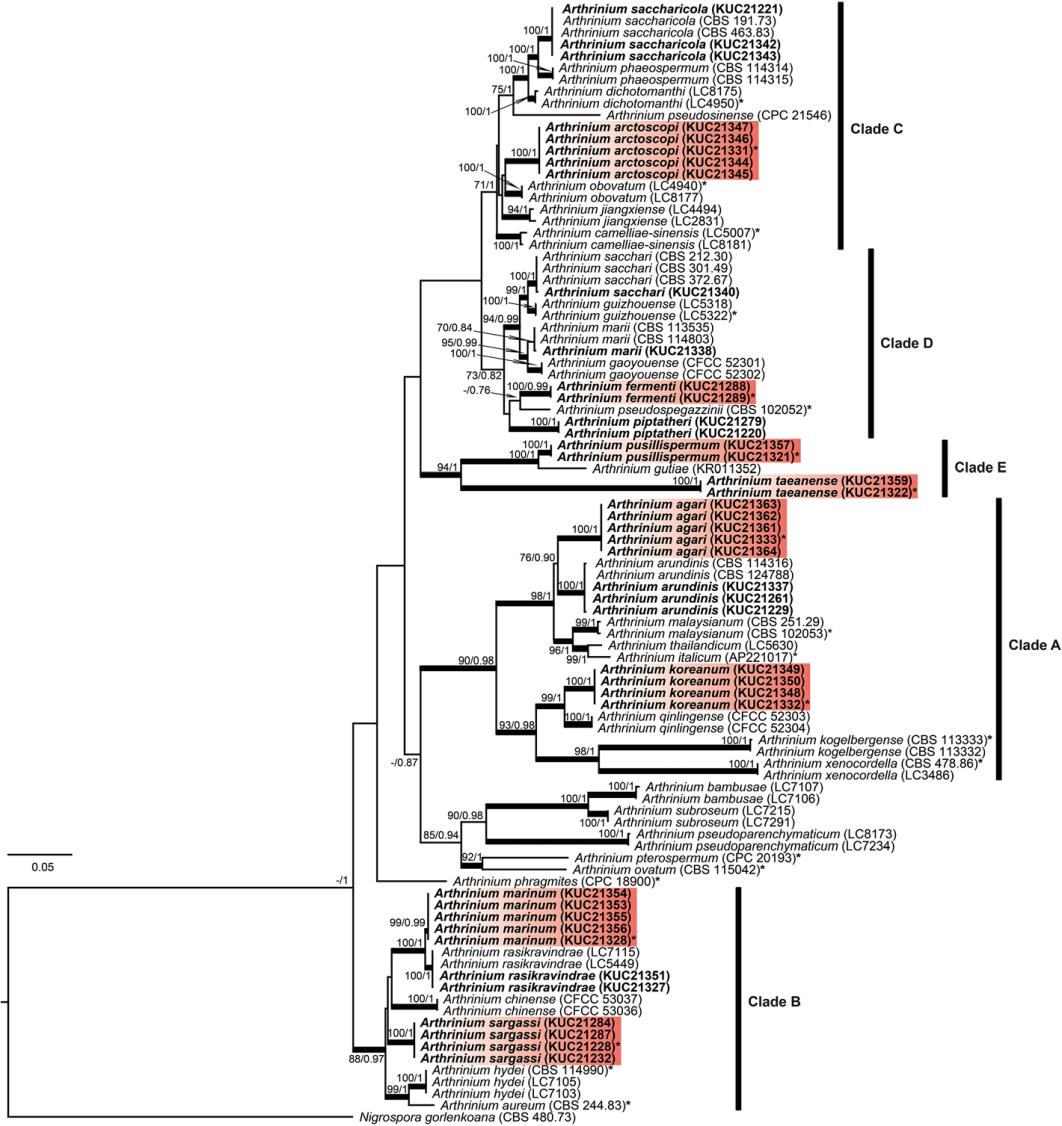
ML tree based on the ITS, TUB, and TEF regions combined. The numbers at the nodes indicate ML bootstrap support (BS) > 75% and Bayesian posterior probabilities (PP) > 0.75 as BS/PP. The thickened branches indicate support greater than 85% for BS and 0.95 for PP. A hyphen (‘-‘) indicates values of BS < 70% or PP < 0.75. Ex-holotype strains are indicated with asterisks (‘*’). The fungal cultures examined in this study are shown in bold. Red boxes indicate the novel species. The numbers in the brackets indicate strain number. The scale bar indicates the nucleotide substitutions per position

A total of 21 ITS (580–1150 bp), 24 TEF (420–970 bp), and 22 TUB (400–560 bp) sequences were newly generated for the 41 *Arthrinium* strains. The ITS phylogeny contained 124 terminals, including *Nigrospora gorlenkoana* as outgroup. The concatenated three-gene phylogeny contained 95 terminals, consisting of 749, 613, and 503 characters respectively, including gaps. Preliminary identification was based on the ITS region, and multigene analysis was used to test the identifications, determine the phylogenetic relationships among the taxa, and to resolve closely related species. Both the ML and Bayesian analyses showed the same tree topologies and the ML tree is represented (Figs. 1, 2).

The 41 *Arthrinium* strains obtained in this study formed five clades (A, B, C, D, and E), both in the ITS-based and combined phylogeny analyses (Figs. 1, 2). In the ITS tree, many *Arthrinium* species were distinguished from one another. However, some were not clearly separated (clades B and D) and the relationships of the others (clades C and D) were not resolved. The above problem was solved in the individual trees of TEF and TUB (Figs. 1S, 2S), and the multigene tree based on the ITS, TUB, and TEF regions (Fig. 2). The multigene analysis supported the conclusion that six taxa corresponded to known species. Eight putatively novel species were classified into five clades (Fig. 2). The eight species were clearly separated from the previously sequenced taxa, each forming a clade with high support (over 99% of BS, 0.99 of PP) (Fig. 2). *Arthrinium agari* and *A. koreanum*. Were included in clade A, *A. piptatheri* and *A. fermenti* were in clade D, and *A. pusillispermum* and *A. taeanense* were in clade E. Comparison with morpho-anatomical and other data of species that have so far not been sequenced supported our interpretation of these eight entities representing novel species.

## TAXONOMY

***Arthrinium agari*** S.L. Kwon, S. Jang & J.J. Kim, **sp. nov.**

MycoBank MB834592

(Fig. 3)

**Fig. 3:**
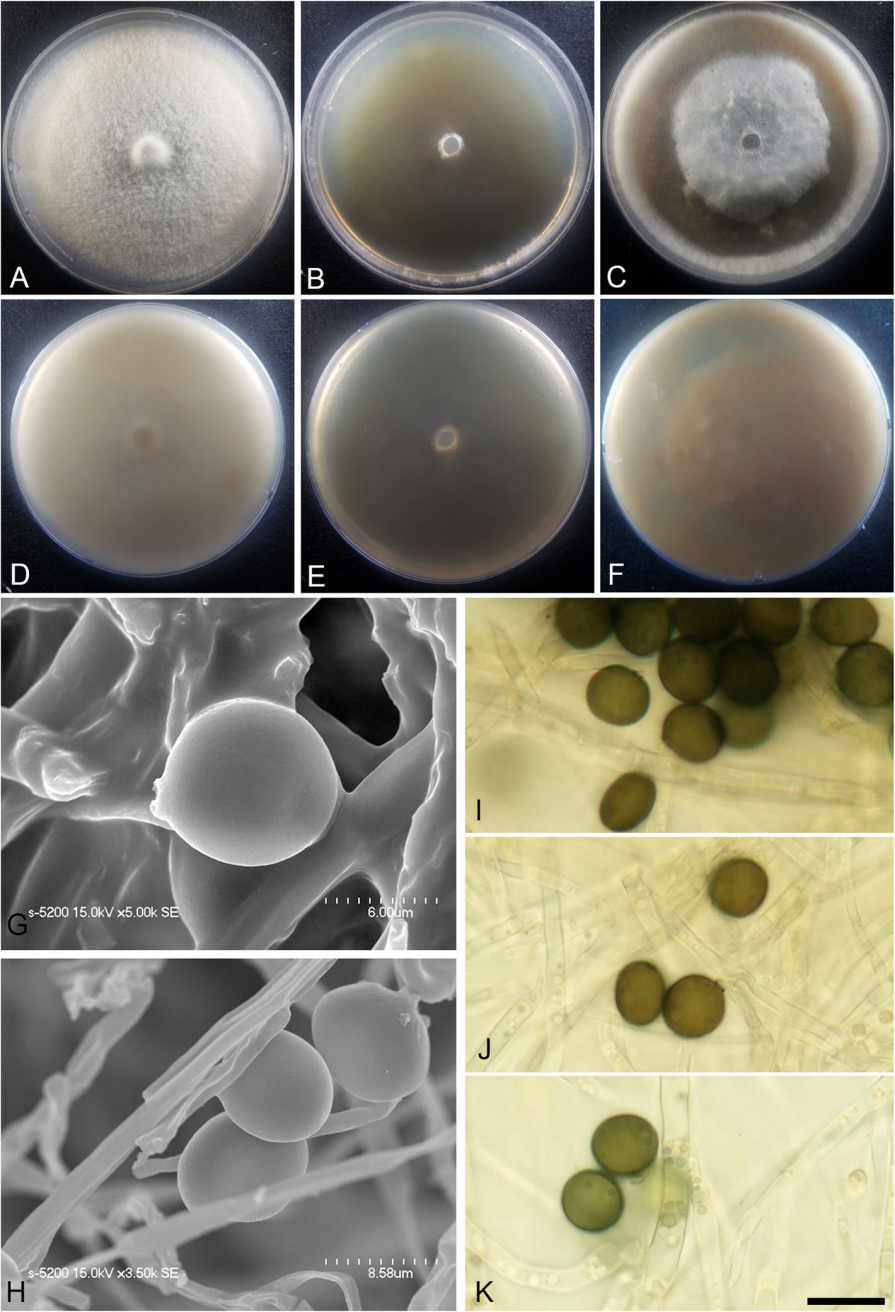
*Arthrinium agari* (KUC21333). **a-c** Colonies on PDA (**a**), MEA (**b**), and OA (**c**) (top); **d-f**, colonies on PDA (**d**), MEA (**e**), and OA (**f**) (bottom); **g-h**, conidia under SEM; **i-k**, conidia attached to conidiogenous cells; scale bar = 10 μm

*Etymology*: ‘*agari*’ refers to the generic name of *Agarum cribrosum*, the source of the type strain.

**Fig. 4:**
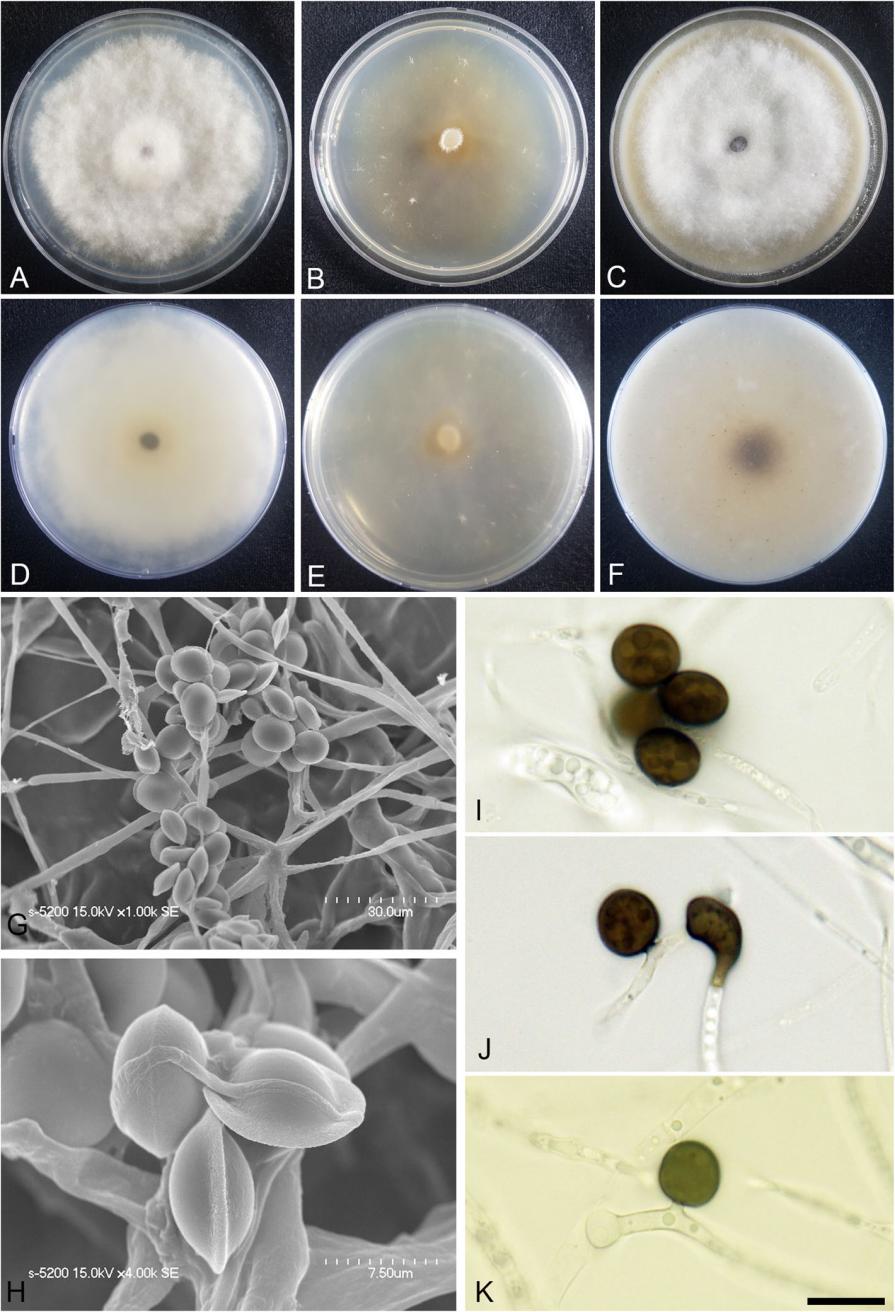
*Arthrinium arctoscopi* (KUC21331). **a-c** Colonies on PDA (**a**), MEA (**b**), and OA (**c**) (top); **d-f**, colonies on PDA (**d**), MEA (**e**), and OA (**f**) (bottom); **g-h**, conidia under SEM; I-K, conidia attached to conidiogenous cells; scale bar = 10 μm

**Fig. 5:**
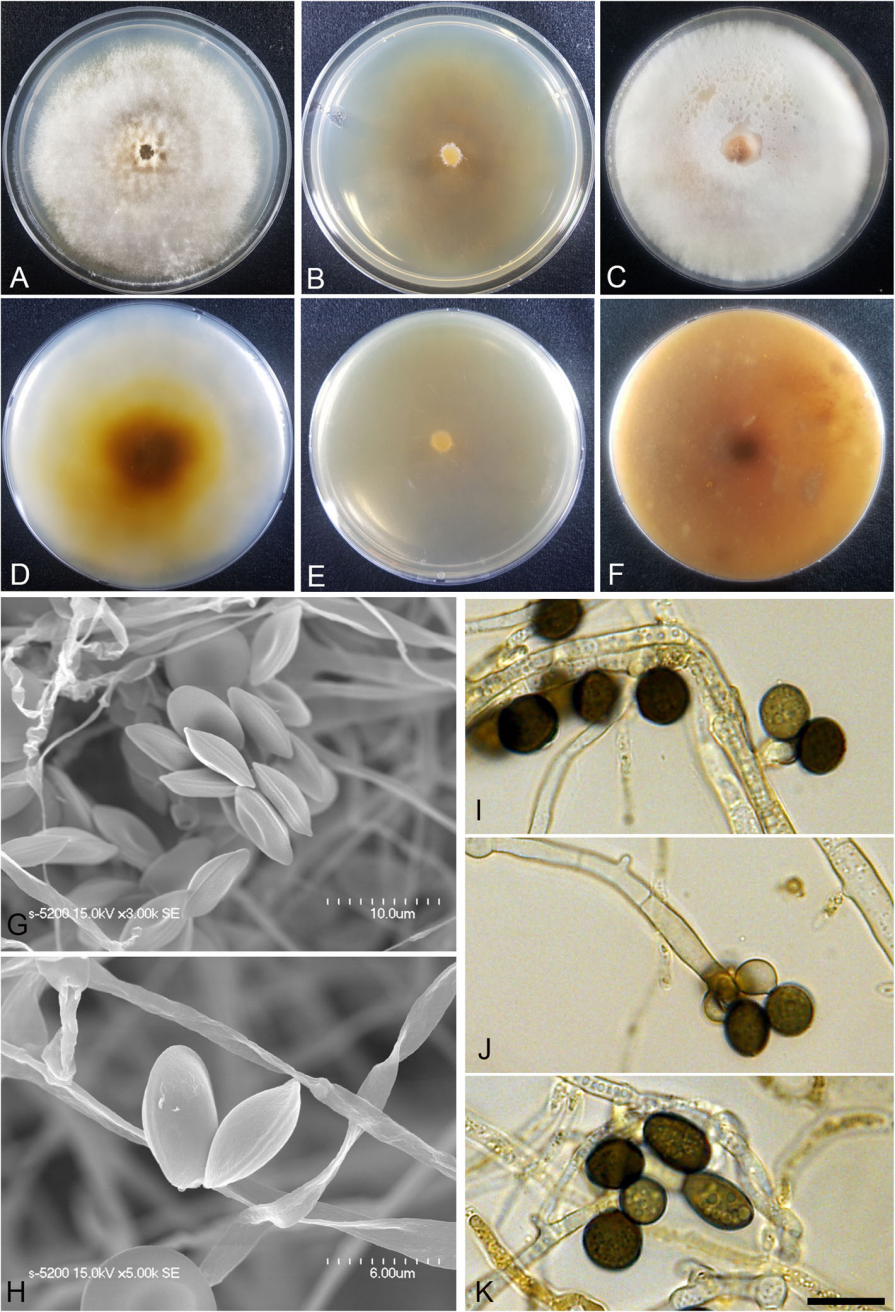
*Arthrinium fermenti* (KUC21288). **a-c**, Colonies on PDA (**a**), MEA (**b**), and OA (**c**) (top); **d-f**, colonies on PDA (**d**), MEA (**e**), and OA (**f**) (bottom), **g-h**, conidia under SEM; **i-k**, conidia attached to conidiogenous cells; scale bar = 10 μm

*Molecular diagnosis*: *Arthrinium agari* is distinguished from the phylogenetically most closely related species, *A. arundinis*, by unique single nucleotide polymorphisms in the three loci used in this study (Figs. 3S, 4S, 5S): ITS positions 21 (C), 31 (indel), 36 (C), 38 (T), 93 (C), 111 (C), 113 (T), 122–124 (indel), 190–203 (indel), 205 (indel), 214–223 (indel), 227 (G), 228 (A), 253 (G), 259 (A), 291 (A), 535 (T), and 645 (indel); TEF positions 14 (A), 16 (G), 17 (T), 32 (C), 35 (A), 47 (C), 54 (T), 59–62 (indel), 64 (T), 65 (T), 79 (G), 85 (G), 96 (T), 125 (G), 135 (indel), 151 (C), 173 (G), 174 (A), 176 (G), 192 (T), 213 (C), 249 (G), 265 (C), 271 (C), 288 (G), 302 (T), 306 (G), 312 (indel), 331 (G), and 494 (A); TUB positions 15 (G), 29 (A), 31 (A), 62 (T), 67 (G), 80 (T), 89 (A), 98 (G), 99 (C), 138 (T), 139 (T), 140 (T), 143 (T), 199 (T), 208 (A), 210 (A), 212 (A), 223 (T), 229 (A), 232 (T), 312 (C), 324 (A), 331 (G), 377 (T), 428 (C), 467 (T), and 482 (A).

*Type*: **Korea:** Gangwon-do, Yangyang-gun, 38°07′04.8″N, 128°38′00.8″E, isolated from *Agarum cribrosum*, 11 Sept. 2016, *M.S. Park* (Herb. KCTC 46909 – holotype preserved in a metabolically inactive state; KUC21333 = NIBRFGC000501588, SFC20161014-M18 – ex-type cultures).

*Description*: *Mycelium* of smooth, hyaline, branched, septate, hyphae 2.0–3.5 μm diam. *Conidiogenous cells* aggregated in clusters on hyphae or solitary, at first hyaline, becoming pale green, cylindrical, sometimes ampulliform. *Conidia* brown, smooth to granular, globose to subglobose in surface view, (8.5–)9.0–10.5 × (7.0–)7.5–8.5 (− 9.0) μm ($\overline{x}$ = 9.5 × 8.1 μm, *n* = 30); lenticular in side view, with equatorial slit, 5.5–7.0 μm wide ($\overline{x}$ = 6.4 μm, *n* = 30), elongated cell observed.

*Culture*: PDA: colonies thick, concentrically spreading with aerial mycelium, margin irregular; mycelia white to grey and pale brown coloured; sporulation on hyphae; dark olive-brown (2.5Y 3/3) pigment diffused in media; odour indistinct. MEA: colonies low, flat, concentrically spreading with sparse aerial mycelium, margin circular; mycelia white; sporulation not observed; pigment absent in medium; odour indistinct. OA: colonies thick, concentrically spreading with aerial mycelium, margin circular; mycelia white to pink; sporulation was not observed; partially pink (2.5YR 8/3) pigment diffused in media; odour indistinct. *Colony diameters* (in mm after 120 h): 15 °C PDA 19–20, MEA 15–18, OA 11–13; 20 °C PDA 34–35, MEA 28–34, OA 20–23; 25 °C PDA 24–28, MEA 22–25, OA 19–20.

*Additional material examined*: **Korea:** Gangwon-do, Yangyang-gun, 38°07′04.8″N, 128°38′00.8″E, isolated from *Agarum cribrosum*, 11 Sept. 2016, *M.S. Park* (KUC21361, KUC21362, KUC21363, and KUC21364).

*Notes*: *Arthrinium agari* is phylogenetically related to *A. arundinis* (over 97.52% similarity in the ITS region, 93.74% in the TEF region, and 93.64% in the TUB region) (Figs. 1, 2). The two species also morphologically resemble each other. The two species have smooth, hyaline, branched, septate mycelium, and ampulliform conidiogenous cells that cluster on hyphae. *Arthrinium arundinis* and *A. agari* have similar conidia shape (brown, globose in surface view, lenticular in side view) (Crous & Groenewald 2013). However, *A. agari* can be distinguished from *A. arundinis* by its larger conidia (*A. agari*: 8.5–10.5 × 7.0–9.0 μm, *A. arundinis*: (5–)6–7 × 3–4 μm diam) (Crous & Groenewald 2013).

*Arthrinium agari* and *A. sinensis* (non-sequenced species) also have similar conidia shape (globose in surface view, lenticular in side view). However, they can be distinguished by the shape of conidiogenous cell; cylindrical and sometimes ampulliform in *A*. *agari*, whereas lageniform in *A. sinensis* (Table 2).

**Table 2:** Summary of conidial morphology of *Arthrinium* species. Newly established species in this study are shown in bold

Species ^***1***^	Habitat ^***2***^	Isolation source	Country ^***3***^	Conidia in surface view		Conidia in side view	
				Shape	Diam (μm)	Shape	Diam (μm)
*A. aureum* ^*A*^	A	Airborn spore	ES	globose	10–30 × 10–15	–	–
*A. guizhouense* ^*b*^	A	Airborn spore	CN	globose to elongate ellipsoid	5–7.5 × 4–7	–	–
*A. mediterranei* ^*e*^	A	Airborn spore	ES	lentiform	9–9.5 × 7.5–9	–	–
*A. serenense* ^*k*^	A	Airborn spore	ES	–	10–11 × 8–9.5	–	–
*A. hispanicum* ^*e*^	M	Beach sand	ES	globose to ellipsoid	7.5–8.5 × 6–7.5	lenticular	6.5
***A. agari***	M	Costariaceae	KR	**globose to elongate ellipsoid**	**8.5–10.5 × 7–9**	**lenticular**	**5.5–7**
***A. arctoscopi***	M	*Egg of Arctoscopus japonicus*	KR	**globose to elongate ellipsoid**	**9.5–13 × 7.5–12**	**lenticular**	**5.5–7.5**
***A. koreanum***	M	*Egg of A. japonicus*	KR	**globose to ellipsoid**	**7.5–11 × 5.5–10**	**lenticular**	**4–6.5**
*A. algicola* ^p*^	M	Sargassaceae	UA	lentiform	10.5–15 × 6–8	–	–
***A. sargassi***	M	Sargassaceae	KR	**globose to elongate ellipsoid**	**8.5–11.5 × 8–11**	**lenticular**	**5.5–7.5**
***A. fermenti***	M	Seaweed	KR	**globose to elongate ellipsoid**	**7.5–9 × 7–9**	**lenticular**	**6–7**
***A. marinum***	M	Seaweed	KR	**globose to elongate ellipsoid**	**9.5–13 × 7.5–10**	**lenticular**	**6–7.5**
***A. pusillispermum***	M	Seaweed	KR	**globose to subglobose, elongate cell**	**4–6.5 × 3–5.5**	**lenticular**	**3.5–4.5**
***A. taeanense***	M	Seaweed	KR	**globose to elongate ellipsoid**	**5–7 × 4–6**	**lenticular**	**4–5**
*A. saccharicola* ^*e*^	M/ P	*Egg of A. japonicus*/ Poaceae	KR/ NL	globose to ellipsoid	(7–)8–9(−10)	lenticular	(4–)5(−6)
*A. sacchari* ^*a*^	M/ P	*Egg of A. japonicus*/ Poaceae	UK/ KR	globose	(6–)7(−8)	lenticular	(3.5–)4
***A. rasikravindrae*** ^***c***^	M/ P	*Egg of A. japonicus*/ Poaceae	KR/ CN	**globose to ellipsoid**	**7–9.5 × 6.5–9**	**lenticular**	**5–6.5**
*A. arundinis* ^*a*^	M/ P	Sargassaceae/ Poaceae	IR/ KR	globose	(5–)6–7	lenticular	3–4
***A. piptatheri*** ^***n***^	M/ P	Sargassaceae/ Poaceae	KR/ ES	**globose to elongate ellipsoid**	**7.5–10 × 7–9**	**lenticular**	**4.5–6**
*A. marii* ^a^	M/ P	Seaweed/ Poaceae	KR/ HK	globose to elongate ellipsoid	8–10(−13)	lenticular	(5–)6(−8)
*A. sporophleum* ^*I*^	P	Poaceae	DE	fusiform	11–14 × 6–8	–	–
*A. descalsii* ^*n*^	P	Poaceae	ES	globose to ellipsoid	(5–)7(−8)	lenticular	6–7
*A. mytilomorphum* ^*b*^	P	Poaceae	IN	fusiform or navicular	20–30 × 6–8.5	–	–
*A. ovatum* ^*a*^	P	Poaceae	HK	oval to boldly ellipsoid	18–20	–	12–14
*A. ibericum* ^*n*^	P	Poaceae	PT	globose to ellipsoid	(9–)10(−12)	lenticular	(6–)7(−8)
*A. italicum* ^*n*^	P	Poaceae	IT, ES	globose	4–6 × 3–4	lenticular	–
*A. hydei* ^*a*^	P	Poaceae	CN	globose	(15–)17–19(−22)	lenticular	11–12
*A. bambusae* ^*b*^	P	Poaceae	CN	subglobose to ellipsoid	11.5–15.5 × 7–14	–	–
*A. jiangxiense* ^*b*^	P	Poaceae	CN	globose to ellipsoid, granular	7.5–10	lenticular	4.5–7
*A. neogarethjonesii* ^*x*^	P	Poaceae	CN	globose to subglobose	20–35 × 15–30	–	–
*A. pseudoparenchymaticum* ^*b*^	P	Poaceae	CN	globose to subglobose	13.5–27 × 12–23.5	–	–
*A. pseudosinense* ^*a*^	P	Poaceae	NL	ellipsoid	8–10 × 7–10	–	7–8
*A. setostromum* ^*z*^	P	Poaceae	CN	subglobose to obovoid	18–20 × 15–19	–	–
*A. subroseum* ^*b*^	P	Poaceae	CN	globose to subglobose, ellipsoid	12–17.5 × 9–16	–	–
*A. thailandicum* ^*i*^	P	Poaceae	CN/ TH	globose to elongate ellipsoid	5–9 × 5–8	lenticular	–
*A. longistromum* ^*i*^	P	Poaceae	TH	asexual morph: Undetermined	–	–	–
*A. neosubglobosa* ^*d*^	P	Poaceae	CN	asexual morph: Undetermined.	–	–	–
*A. subglobosa* ^*h*^	P	Poaceae	TH	asexual morph: Undetermined.	–	–	–
*A. macrosporum* ^E*^	P	Poaceae	CN	–	17–27	–	–
*A. paraphaeospermum* ^*j*^	P	Poaceae	TH	globose to ellipsoid	10–19	lenticular	–
*A. hyphopodii* ^*h*^	P	Poaceae	TH	globose to subglobose	5–10 × 4–8	–	–
*A. chinense* ^*s*^	P	Poaceae	CN	subglobose to lenticular	8.5–12 × 5.5–9	–	–
*A. qinlingense* ^*l*^	P	Poaceae	CN	globose to suborbicular	5–8	–	–
*A. phaeospermum* ^*a*^	P	Poaceae	IR, KR	globose to ellipsoid	(9–)10(−12)	lenticular	6–7
*A. gaoyouense* ^*l*^	P	Poaceae	CN	globose to elongate ellipsoid	5–8	lenticular	4–8
*A. phragmitis* ^*a*^	P	Poaceae	IT	ellipsoid to ovoid	9–10(−12)	lenticular	(5–)6(−7)
*A. esporlense* ^*n*^	P	Poaceae	ES	globose	(8–)9–12(−13)	lenticular	6–8
*A. hysterinum* ^*n*^	P	Poaceae	ES	globose to obovoid	15–21 × 14–19	–	–
*A. phyllostachydis* ^*y*^	P	Poaceae	CN	globose to subglobose, oval or irregular	5–6 × 4–6	–	–
*A. yunnanum* ^*i*^	P	Poaceae	CN	globose to obovoid	17.5–26.5 × 15.5–25	–	–
*A. spegazzinii* ^t*^	P	Poaceae	AR	clavate, oval or elliptical	5–8 × 3–6	–	–
*A. euphorbiae* ^*f*^	P	Poaceae	BD	circular or nearly circular	4–5.5 × 3–4	lenticular	–
*A. lobatum* ^t*^	P	Poaceae	VE	oval or broadly ellipsoid	17–20 × 12–14	–	–
*A. balearicum* ^*n*^	P	Poaceae	ES	asexual morph: Undetermined.	–	–	–
*A. garethjonesii* ^*d*^	P	Poaceae	CN	asexual morph: Undetermined.	–	–	–
*A. sinensis* ^H*^	P	Arecaceae	CN	rounded (conidiogenous cell lageniform)	9–12	lenticular	6–8
*A. trachycarpum* ^*w*^	P	Arecaceae	CN	subglobose to elongate ellipsoid	6–8.5 × 4–6	–	–
*A. locutum-pollinis* ^*v*^	P	Brassicaceae	CN	globose to elongate ellipsoid	8–15 × 5–9.5	–	–
*A. camelliae-sinensis* ^*b*^	P	Brassicaceae, Theaceae	CN	globose to subglobose	9–13.5 × 7–12	–	–
*A. caricicola* ^*r*^	P	Cyperaceae	DE	Ultimately cigar or diatom-shape	42–47 × 9–12	–	–
*A. carinatum* ^D*^	P	Cyperaceae	DE	irregular shape	–	–	–
*A. sporophleoides* ^r*^	P	Cyperaceae	AU, DE	fusiform	11–14 × 5–5.5	polygonal	–
*A. austriacum* ^n*^	P	Cyperaceae	AU	irregularly polygonal or rounded	9–12	polygonal	8–10
*A. fuckelii* ^n, I*^	P	Cyperaceae	NO	quadrangular	11–16 × 11–16 × 5–9	–	–
*A. globosum* ^n*^	P	Cyperaceae	FI	globose or almost round	8–10 × 7–9	–	–
*A. japonicum* ^*u*^	P	Cyperaceae	JP	fusiform, navicular	38–56 × 14–20	–	–
*A. kamtschaticum* ^u*^	P	Cyperaceae	RU	broadly U-shape with ends rounded	22–32 × 10–14	–	–
*A. minus* ^*n*^	P	Cyperaceae	DE	curved, rounded at the ends	9–10 × 6–7	–	–
*A. morthieri* ^F*^	P	Cyperaceae	CH	ovoid, subglobose, granular, rounded tips	18–20 × 4–7	–	–
*A. muelleri* ^n*^	P	Cyperaceae	CH	curved conidia	15–20 × 8–10	–	–
*A. naviculare* ^n*^	P	Cyperaceae	FI	irregular shape	–	–	–
*A. puccinioides* ^*n, r*^	P	Cyperaceae	FR	polygonal with rounded angles	9–11 × 8–9	–	–
*A. sporophlaeum* ^r*^	P	Cyperaceae, Juncaecae	PT	broadly ovate to lemon-shaped	7–12 × 6–8	–	5–8
*A. pterospermum* ^*a*^	P	Cyperaceae	AU, NZ	finely roughened irregular	15–25	–	8–10
*A. cuspidatum* ^*r*^*,* ^C*^	P	Cyperaceae, Juncaecae	CA, IN, US, ZA	horn-like tips (tips size: 7 μm)	21.5 × 10	–	–
*A. jatrophae* ^*f*^	P	Euphorbiaceae	IN	spherical	6.5–9.5	lenticular	3–6.5
*A. pseudospegazzinii* ^*a*^	P	Euphorbiaceae	MY	globose	(7–)8–9	lenticular	5–6
*A. obovatum* ^*b*^	P	Fagaceae	CN	obovoid, elongated to ellipsoidal	11–16.5/ 16–31 × 9–16	–	–
*A. gutta* ^B*^	P	Fagaceae	IT	drop-shaped, oval	9–12 × 7–11	–	–
*A. sphaerospermum* ^r, t*^	P	Iridaceae, Myrtaceae*,* Poaceae	FR	spherical or subspherical	7–8	–	–
*A. ushuvaiense* ^r*^	P	Juncaceae	AR	fusiform or navicular	17–25 × 6–9	–	–
*A. luzulae* ^t*^	P	Juncaceae	CH	curved with horn-like tips	18–21 × 12–14	–	8–11
*A. malaysianum* ^*a*^	P	Lauraceae, Euphorbiaceae	MY	globose	5–6	lenticular	3–4
*A. kogelbergense* ^*a*^	P	Restionaceae	ZA	globose to ellipsoid	9–10 × 7–8	lenticular	4–5
*A. dichotomanthi* ^*b*^	P	Rosaceae	CN	globose to subglobose	9–15 × 6–12	lenticular	–
*A. vietnamense* ^*o*^	P	Rutaceae	VN	globose	5–6	–	3–4
*A. xenocordella* ^*a*^	P	Theaceae	CN	globose to somewhat ellipsoid	9–10	lenticular	6–7
*A. aquaticum* ^*q*^	P	unknown	CN	globose to subglobose	9–11 × 8–10	–	–
*A. scriptum* ^G*^	P	unknown	DE	egg-shape, pear-shape	–	–	–
*A. urticae* ^*r*^	P	unknown	IN, TR, CU, BE	subspherical	4–6 × 3–4	–	–
*A. gutiae* ^*g*^	I	Gut of a grasshopper	IN	globose	4.5–6.0	lenticular	2–6
A. leucospermum^*^	–	–	–	–	–	–	–

^*1*^The reference species were cited from the following marks: ^*a*^ (Crous and Groenewald 2013), ^*b*^ (Wang et al. 2018), ^*c*^ (Singh et al. 2013), ^*d*^ (Dai et al. 2016), ^*e*^ (Larrondo 1992), ^*f*^ (Sharma et al. 2014), ^*g*^ (Crous et al. 2015), ^*h*^ (Senanayake et al. 2015), ^*i*^ (Dai et al. 2017), ^*j*^ (Hyde et al. 2016), ^*k*^ (Larrondo and Calvo 1990), ^*l*^ (Jiang et al. 2018), ^*m*^ (Wang et al. 2017), ^n^ (Pintos et al. 2019), ^o^ (Wang et al. 2017), ^*p*^ (Jones et al. 2009), ^*q*^ (Luo et al. 2019), ^*r*^ (Cooke 1954), ^s^ (Jiang et al. 2020), ^*t*^ (Ellis 1972), ^*u*^ (Pollack and Benjamin, 2020), ^*v*^ (Zhao et al. 2018), ^*w*^ (Yan et al. 2019), ^*x*^ (Hyde et al. 2020), ^*y*^ (Yang et al. 2019), ^*z*^ (Jiang et al. 2019), ^*A*^ (Calvo 1980), ^*B*^ (Rambelli et al. 2008), ^*C*^ (Sukova 2004), ^*D*^ (Harvard University Herbaria and Libraries (HUH), n.d.), ^*E*^ (Joint Publications Research Service Arlington (JPRSA) VA, 1977), ^*F*^ (Fungi of Great Britain and Ireland (FGBI), n.d.), ^*G*^ (Rabenhorst and Lindau 1907), ^*H*^ (Hyde et al. 1998), and ^*I*^ (Minter and Cannon 2018). The species which not have any information about ITS, TEF, and TUB regions were marked by “*”. Sequenced species were presented with GenBank accession numbers in supplementary Table 1S. ^*2*^Habitats were indicated by following abbreviation: A, Air; M, Marine; M/P, Marine and Plant; P, Plant. ^*3*^Country is presented by standard defining code (ISO 3166-1alpha-2) for the names of country

***Arthrinium arctoscopi*** S.L. Kwon, S. Jang & J.J. Kim, **sp. nov.**

MycoBank MB834593

(Fig. 4)

*Etymology*: ‘*arctoscopi’* refers to the generic name of *Arctoscopus japonicus*, the substrate of on which it was found.

*Molecular diagnosis*: *Arthrinium arctoscopi* is distinguished from phylogenetically most closely related species, *A. obovatum*, by unique single nucleotide polymorphisms in the three loci used in this study (Figs. 3S, 4S, 5S): ITS positions 112–124 (indel), 128–137 (indel), 190 (indel), 192 (G), 223 (T), 225 (indel), 226 (indel), 253–254 (indel), 618 (G), 621 (C), 624 (C), and 651 (G); TEF positions 32 (T), 33 (T), 76 (G), 131 (G), 132 (C), 145 (T), 148–150 (indel), 207 (indel), 208 (T), 210 (T), 211 (T), 269 (G), 304 (A), 305 (C), 316 (C), 320 (C), 324 (A), 328 (T), and 333 (A); TUB position 5 (T), 8 (C), 27 (G), 38 (T), 53 (G), 62 (A), 68 (C), 79 (C), 80 (A), 82 (G), 87 (T), 90 (A), 106 (A), 112 (T), 144 (A), 211 (indel), 212 (T), 225 (T), 227 (C), 311 (T), 334 (T), 467 (C), 479 (C), and 506 (C).

*Type*: **Korea:** Gangwon-do, Goseong-gun, 38°28′44.0″N, 128°26′18.9″E, isolated from Egg masses of *Arctoscopus japonicus*, 10 Nov. 2016, *M.S. Park* (Herb. KCTC 46907 – holotype preserved in a metabolically inactive state; KUC21331 = NIBRFGC000501586, SFC20200506-M05 –ex-type cultures).

*Descriptions*: *Mycelium* of smooth, hyaline, branched, septate, hyphae 2.5–4.0 μm diam. *Conidiogenous cells* aggregated in clusters on hyphae or solitary, at first hyaline, becoming pale green, cylindrical, sometimes ampulliform. *Conidia* brown, smooth to granular, globose to elongate ellipsoid in surface view, (9.5–)10–12 (− 13) × (7.5–)8.0–11 (− 12) μm ($\overline{x}$ = 11.1 × 10 μm, *n* = 30); lenticular in side view, with equatorial slit, 5.5–7.5 μm wide ($\overline{x}$ = 6.5 μm, *n* = 30), elongated cell observed.

*Culture*: PDA: colonies thick, concentrically spreading with aerial mycelium, margin irregular; mycelia creamy white; sporulation was not observed; pigment absent in medium; odour indistinct. MEA: colonies flat, concentrically spreading with aerial mycelium, margin irregular; mycelia white; sporulation on hyphae after 2 weeks, spores black; pigment absent in medium; odour indistinct. OA: colonies thick, concentrically spreading with aerial mycelium, margin irregular; mycelia creamy pale yellow; sporulation not observed; very dark greyish brown (2.5Y 3/2) pigment diffused from centre into medium; odour indistinct. *Colony diameters* (in mm after 120 h): 15 °C PDA 9, MEA 13–15, OA 11–13; 20 °C PDA 18–24, MEA 18–22, OA 14–18; 25 °C PDA 5–7, MEA 4–5, OA 7–9.

*Additional material examined*: **Korea:** Gangwon-do, Goseong-gun, 38°28′44.0″N, 128°26′18.9″E, isolated from egg masses of *Arctoscopus japonicus*, 10 Nov. 2016, *M.S. Park* (KUC21344, KUC21345, KUC21346, and KUC21347).

*Notes*: *Arthrinium arctoscopi* is closely related to *A. obovatum* (98.84% similarity in the ITS region, 96.10% in the TEF region, and 94.31% in the TUB region) and *A. aquaticum* (99.80% similarity in the ITS region). However, *A. arctoscopi* can be distinguished from *A. obovatum* by the conidial shape and growth rate; the conidia of *A. arctoscopi* are globose to subglobose, whereas those of *A. obovatum* are obovoid or occasionally elongated to ellipsoid in shape (Wang et al. 2018). In addition, the growth rate of *A. arctoscopi* (7–9 mm in 7 d at 25 °C, PDA) is slower than that of *A. obovatum* (covering a 90 mm Petri dish in 7 d at 25 °C, PDA) (Wang et al. 2018). The conidial shape of *A. arctoscopi* is also slightly different from that of *A. aquaticum* (globose to subglobose conidia, 9–11 × 8–10 μm, $\overline{x}$ = 10 × 9 μm, *n* = 20). Two non-sequenced species, *A. algicola* and *A. sinensis*, are morphologically similar to *A. arctoscopi*. The longer length and narrower width of *A. algicola* conidia (10.5–15 × 6–8 μm) and lageniform conidiogenous cell of *A. sinensis* distinguish them from *A. arctoscopi* (Table 2).

***Arthrinium fermenti*** S.L. Kwon, S. Jang & J.J. Kim, **sp. nov.**

MycoBank MB834594

(Fig. 5)

*Etymology*: ‘*fermenti’* refers to the yeast-like odour of the cultures.

*Molecular diagnosis*: *Arthrinium fermenti* is distinguished from the phylogenetically most closely related species, *A. pseudospegazzinii*, by unique single nucleotide polymorphisms in the three loci used in this study (Figs. 3S, 4S, 5S): ITS positions 32 (C), 43 (T), 81 (C), 283 (T), 318 (T), 567 (A), and 644 (indel); TEF positions 9 (C), 35 (C), 44 (A), 67 (A), 81–82 (indel), 84 (indel), 87 (C), 92 (G), 93 (A), 114 (G), 126 (C), 133 (T), 134 (G), 140 (T), 154 (G), 170 (C), 171 (T), 172 (T), 178 (indel), 181 (indel), 192 (C), 206 (indel), 208–211 (indel), 213 (T), 239 (G), 243 (T), 252 (A), 264 (C), 288 (G), 305 (C), 311 (C), 322 (indel), 330 (A), 337 (T), 357 (G), 367 (T), 375 (T), 392 (A), and 473 (T); TUB positions 1 (T), 9 (T), 18–22 (indel), 28 (A), 33 (C), 41 (G), 67 (A), 80 (A), 94 (G), 106 (T), 117 (T), 223 (A), 233 (T), 308 (A), 309 (T), 322 (T), 327 (C), 329 (C), 331 (C), 425 (C), and 437 (T).

*Type*: **Korea:** Jeollanam-do, Haenam-gun, 34°26′07.2″N, 126°28′16.5″E, isolated from seaweed, 23 Apr. 2014, *M.S. Park* (Herb. KCTC 46903 – holotype preserved in a metabolically inactive state; KUC21289 = NIBRFGC000501584, SFC20140423-M86 – ex-type cultures).

*Description*: *Mycelium* of smooth, hyaline, branched, septate, 2.0–4.0 μm diam. *Conidiogenous cells* aggregated in clusters on hyphae, at first hyaline, becoming pale brown, polyblastic, discrete, erect, ampulliform. *Conidia* brown, smooth to granular, globose to elongated ellipsoid, (7.5–)8.0–9.0 × 7.0–8.5 (− 9) μm ($\overline{x}$ = 8.32 × 7.4 μm, *n* = 30); lenticular in side view, with equatorial slit, 6.0–7.0 μm wide ($\overline{x}$ = 6.6 μm, *n* = 30).

*Culture*: PDA: colonies thick, concentrically spreading with aerial mycelium, margin irregular; mycelia white to yellow, becoming pinkish to orange after 2 weeks; sporulation on hyphae, spores black; dark reddish brown (5YR 2.5/2) to yellow (2.5Y 8/8) pigment diffused from centre into media; odour strong baker’s yeast-like. MEA: colonies low, flat, concentrically spreading, thin, margin circular; mycelia white; sporulation was not observed; medium reverse with yellow pigment after 2 weeks; odour strong baker’s yeast–like. OA: colonies thick, concentrically spreading with aerial mycelium, margin irregular; mycelia at first white, reverse randomly pale pink to red-grape and pale yellow to brown after 2 weeks; sporulation on hyphae, spores black; dark yellowish brown (10YR 3/4, 3/6) to dark reddish brown (2.5YR 2.5/4) pigment diffused into the medium; odour strong baker’s yeast–like. *Colony diameters* (in mm after 120 h): 15 °C PDA 17, MEA 17–18, OA 13–16; 20 °C PDA 27–30, MEA 21–27, OA 15–18; 25 °C PDA 21–23, MEA 18–19, OA 14–16.

*Additional material examined*: **Korea:** Jeollanam-do, Haenam-gun, 34°26′07.2″N, 126°28′16.5″E, isolated from seaweed, 23 Apr. 2014, *M.S. Park* (KUC21288).

*Notes*: *Arthrinium fermenti* is closely related to *A. pseudospegazzinii* (98.96% similarity in the ITS region, 92.47% in the TEF region, and 95.00% in the TUB region) (Figs. 1, 2). It can be distinguished from the latter by conidial shape and colony colour. The conidia of *A. fermenti* are globose to elongate-ellipsoid, whereas *A. pseudospegazzinii* has uniformly globose conidia (Crous & Groenewald 2013). Moreover, while the colonies of *A. pseudospegazzinii* were light orange on PDA and dirty white with an olivaceous grey patch on OA and MEA (Crous & Groenewald 2013), *A. fermenti* colonies had a yellowish to reddish colour on OA and MEA and a strong yeast odour. *Arthrinium globosum* (non-sequenced species) has a conidia shape similar to that of *A. fermenti* – globose to subglobose. However, a lenticular shape in side view was not observed in *A. globosum* (Table 2).

***Arthrinium koreanum*** S.L. Kwon, S. Jang & J.J. Kim, **sp. nov.**

MycoBank MB834596

(Fig. 6)

**Fig. 6:**
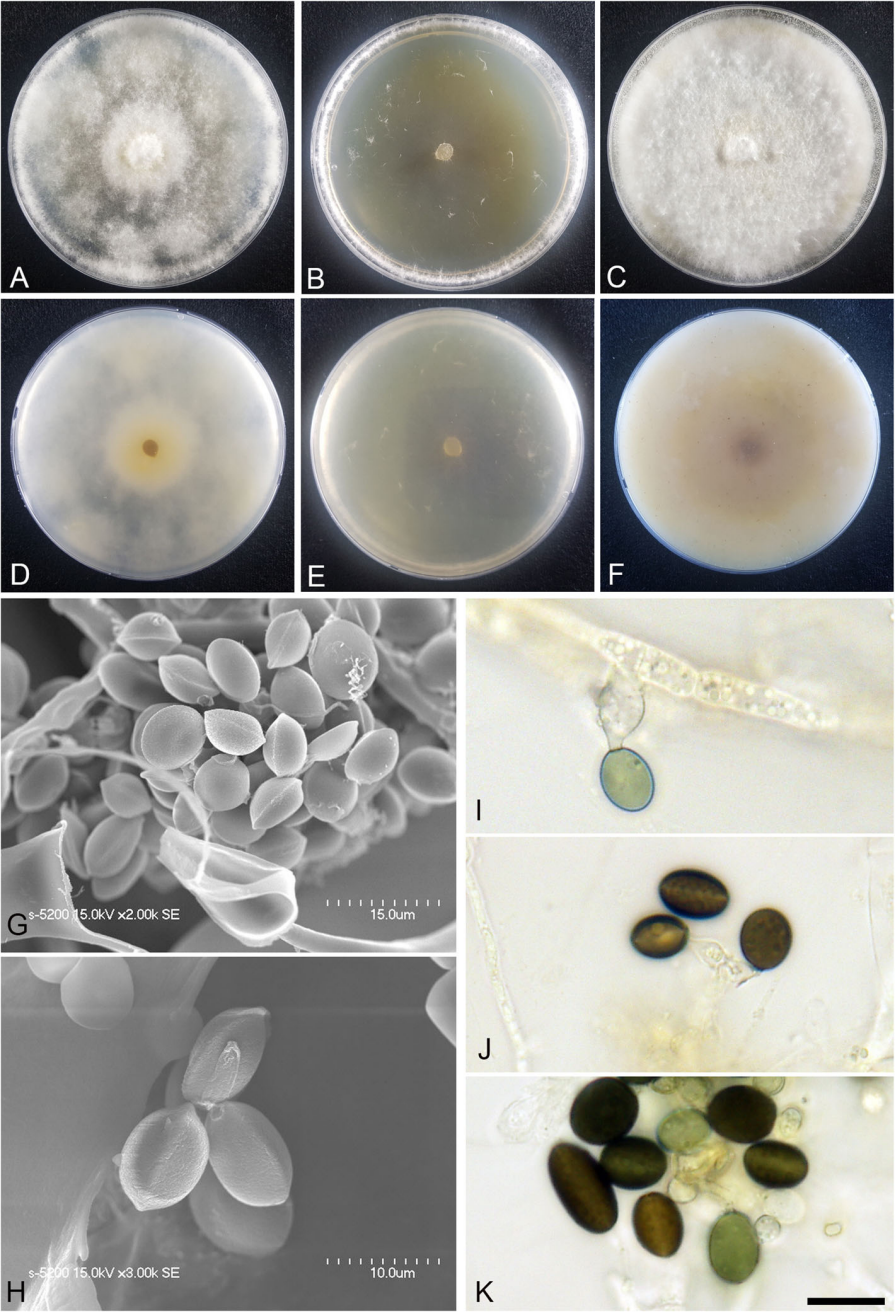
*Arthrinium koreanum* (KUC21332). A-C, Colonies on PDA (**a**), MEA (**b**), and OA (**c)** (top); **d-f**, colonies on PDA (**d**), MEA (**e**), and OA (**f**) (bottom); **g-h**, conidia under SEM; **i-k**, conidia attached to conidiogenous cells; scale bar = 10 μm

*Etymology*: ‘*koreanum*’ refers to the country in which the type locality is located.

*Molecular diagnosis*: *Arthrinium koreanum* is distinguished from the phylogenetically most closely related species, *A. qinlingense*, by unique single nucleotide polymorphisms in the three loci used in this study (Figs. 3S, 4S, 5S): ITS positions 80 (C), 92 (C), 245 (G), 250 (A), 253 (C), 258 (C), 274 (C), and 293 (G); TEF positions 16 (G), 43 (T), 44 (T), 91 (T), 94 (T), 133 (C), 135 (indel), 149 (indel), 152 (T), 153 (C), 154 (A), 156 (C), 157 (A), 161 (T), 162 (C), 199 (indel), 200 (T), 248 (A), 250 (G), 251 (T), 252 (G), 253 (C), 321 (C), 322 (A), 407 (C); TUB positions 4 (G), 5 (T), 18 (A), 38 (T), 49 (A), 64 (T), 68 (G), 78 (A), 80 (G), 89 (G), 98 (C), 113 (G), 114 (G), 199 (C), 309 (G), 326 (A), 410 (C), 413 (C), and 497 (T).

*Type*: **Korea:** Gangwon-do, Goseong-gun, 38°28′44.0″N, 128°26′18.9″E, isolated from egg masses of *Arctoscopus japonicus*, 10 Nov. 2016, *M.S. Park* (Herb. KCTC 46908 – holotype preserved in a metabolically inactive state; KUC21332 = NIBRFGC000501587, SFC20200506-M06 – ex-type cultures).

*Description*: *Mycelium* consisting of smooth, hyaline, branched, septate, hyphae 1.5–6.0 μm diam. *Conidiogenous cells* aggregated in clusters on hyphae, hyaline, cylindrical. *Conidia* brown, smooth to granular, globose to ellipsoid in surface view, (7.5–)8.0–10 (− 11) × (5.5–)6.5–9.5 (− 10) μm ($\overline{x}$ = 9.1 × 8.1 μm, *n* = 30); lenticular in side view, with equatorial slit, 4.0–6.5 μm wide ($\overline{x}$ = 5.3 μm, *n* = 30).

*Culture*: PDA: colonies thick, concentrically spreading with aerial mycelium, margin irregular; mycelia white to pale yellow; sporulation not observed; olive-yellow (2.5Y 6/8) pigment diffused into medium; odour indistinct. MEA: colonies flat, concentrically spreading with sparse aerial mycelium, margin circular; mycelia white; sporulation on hyphae after 2 weeks, spores black; pigment absent in medium; odour indistinct. OA: colonies thick, concentrically spreading with aerial mycelium, margin irregular; mycelia white to orange; sporulation not observed; dark reddish brown (5YR 4/6) pigment diffused in media; odour indistinct. *Colony diameters* (in mm after 120 h): 15 °C PDA 17–18, MEA 15–19, OA 16–17; 20 °C PDA 27–31, MEA 20–23, OA 27–28; 25 °C PDA 6–7, MEA 3–6, and OA 4–5.

*Additional material examined*: **Korea:** Gangwon-do, Goseong-gun, 38°28′44.0″N, 128°26′18.9″E, isolated from egg masses of *Arctoscopus japonicus*, 10 Nov. 2016, *M.S. Park* (KUC21348, KUC21349, and KUC21350).

*Notes*: *Arthrinium koreanum* is closely related to *A. qinlingense* (98.48% similarity in the ITS region, 94.92% in the TEF region, and 94.85% in the TUB region) (Figs. 1, 2). They can be distinguished by their conidial sizes; 7.5–11 × 5.5–10 μm in *A. koreanum* vs. 5–8 μm in diameter in *A. qinlingense* (Jiang et al. 2018). *Arthrinium koreanum* has a similar conidia shape to that of the two non-sequenced species, *A. globosum* and *A. sphaerospermum*. However, the conidia of the latter two species only have globose to subglobose shape, and lenticular shape is not observed in side view (Table 2).

***Arthrinium marinum*** S.L. Kwon, S. Jang & J.J. Kim, **sp. nov.**

MycoBank MB834595

(Fig. 7)

**Fig. 7:**
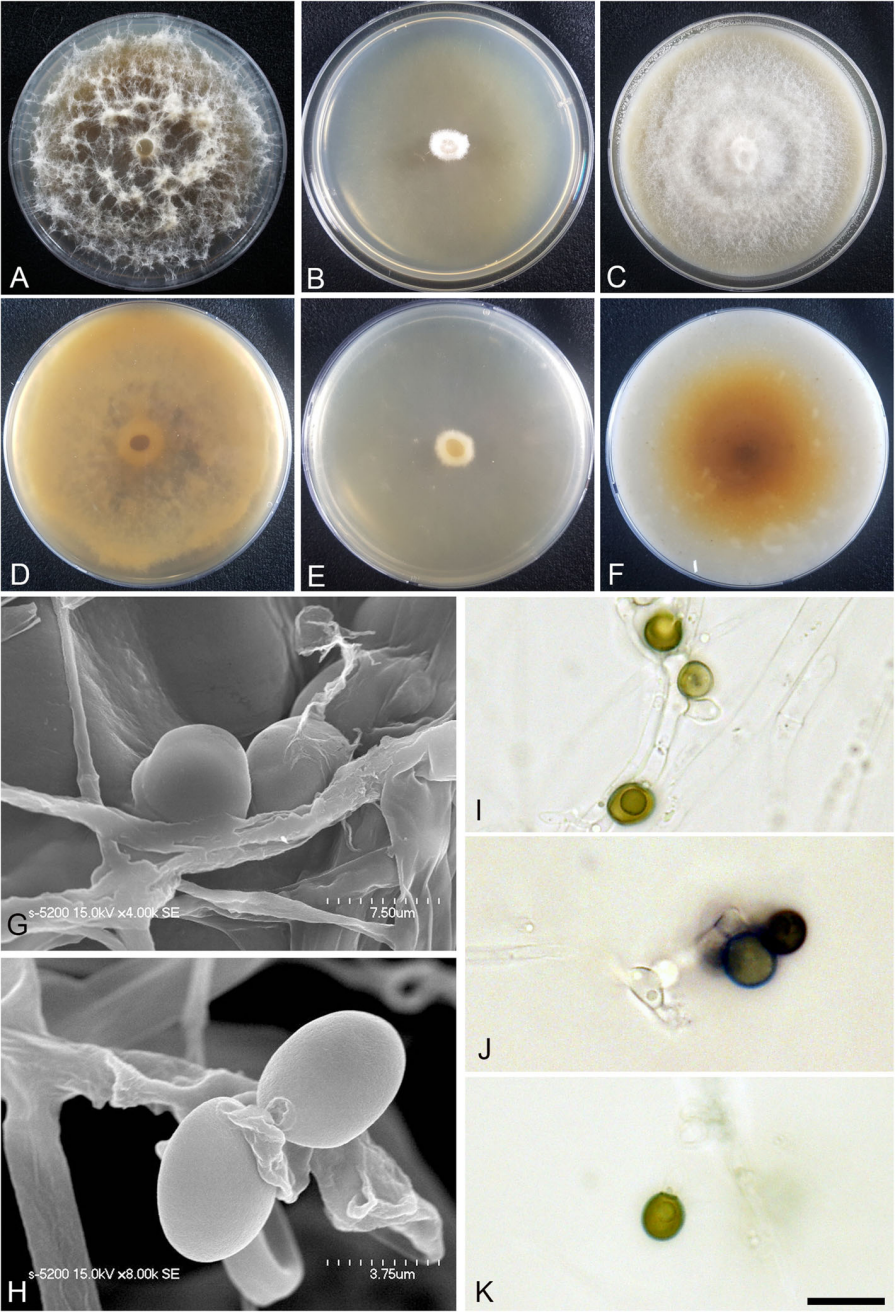
*Arthrinium marinum* (KUC21328). **a-c**, Colonies on PDA (**a**), MEA (**b**), and OA (**c**) (top); **d-f**, colonies on PDA (**d**), MEA (**e**), and OA (**f**) (bottom); **g-h**, conidia under SEM; **i-k** conidia attached to conidiogenous cells; scale bar = 10 μm

*Etymology*: ‘*marinum*’ refers to the marine origin.

*Molecular diagnosis*: *Arthrinium marinum* is distinguished from the phylogenetically most closely related species, *A. rasikravindrae*, by unique single nucleotide polymorphisms in the three loci used in this study (Figs. 3S, 4S, 5S): ITS positions 100% similarity; TEF positions 191 (T), 253 (C), 256 (A), 319 (A), and 372 (C); TUB positions 2 (T), 15 (A), 20 (G), 30 (C), 69 (G), 111 (indel), 314 (G), 363 (T), 437 (C), and 443 (C).

*Type*: **Korea:** Jeollanam-do, Suncheon-si, 34°50′46.9″N, 127°31′31.4″E, isolated from seaweed, 23 Apr. 2014, *M.S. Park* (Herb. KCTC 46905 – holotype preserved in a metabolically inactive state; KUC21328 = NIBRFGC000501583, SFC20140423-M02 –ex-type cultures).

*Description*: *Mycelium* superficial, composed of smooth, hyaline, branched, septate, 3.5–6.0 μm diam. Hyphae. *Conidiogenous cells* aggregated in clusters on hyphae or solitary, hyaline, erect, ampulliform. *Conidia* brown, smooth to granular, globose to elongate ellipsoid in surface view, (9.5–)10–12 (− 13) × (7.5–)8.0–10 μm ($\overline{x}$ = 11.1 × 9.4 μm, *n* = 30); lenticular in side view, with equatorial slit, 6.0–7.5 μm wide ($\overline{x}$ = 7.1 μm, *n* = 30).

*Culture*: PDA: colonies thick and dense, concentrically spreading, margin irregular; mycelia white to pale yellow; sporulation was not observed; pale yellow (5Y 8/4) pigment diffused into medium; odour indistinct. MEA: colonies low, flat, concentrically spreading with sparse aerial mycelium, margin circular; mycelia white colored; sporulation on hyphae around centre after 2 weeks, spores black; pigment absent in medium; odour indistinct. OA: colonies thick, concentrically spreading with aerial mycelium, margin circular; mycelia white to pale yellow; sporulation not observed; yellow to pale green (2.5Y 5/6) pigment diffused into medium; odour indistinct. *Colony diameters* (in mm after 120 h): 15 °C PDA 7–9, MEA 6–12, OA 4–5; 20 °C PDA 16–17, MEA 14–21, OA 7–9; 25 °C PDA 35–47, MEA 32–35, and OA 28–32.

*Additional material examined*: **Korea:** Jeollanam-do, Suncheon-si, 34°50′46.9″N, 127°31′31.4″E, isolated from seaweed, 23 Apr. 2014, *M.S. Park* (KUC21353, KUC21354, KUC21355, and KUC21356).

*Notes*: Although *Arthrinium marinum* and *A. rasikravindrae* were not distinguished on ITS alone (100% similarity in the ITS region), these species formed two distinct clades based on the combined analysis of the ITS, TUB, and TEF regions (99.08% in the TEF region and 97.97% in the TUB region) (Figs. 1, 2). They can also be distinguished by their growth rates: *A. marinum* (16–17 mm in 5 d on PDA at 20 °C) had a slower growth rate than *A. rasikravindrae* KUC21327 (34–39 mm in 5 d on PDA at 20 °C).

Non-sequenced species, *Arthrinium algicola*, has a very similar conidia shape to that of *A. marinum*, However, they are distinguished by the conidia size; 10.5–15 × 6–8 μm in *A. algicola* and (9.5–)10–12(− 13) × (7.5–)8–10 μm in *A. marinum* (Table 2).

***Arthrinium pusillispermum*** S.L. Kwon, S. Jang & J.J. Kim, **sp. nov.**

MycoBank MB834597

(Fig. 8)

**Fig. 8:**
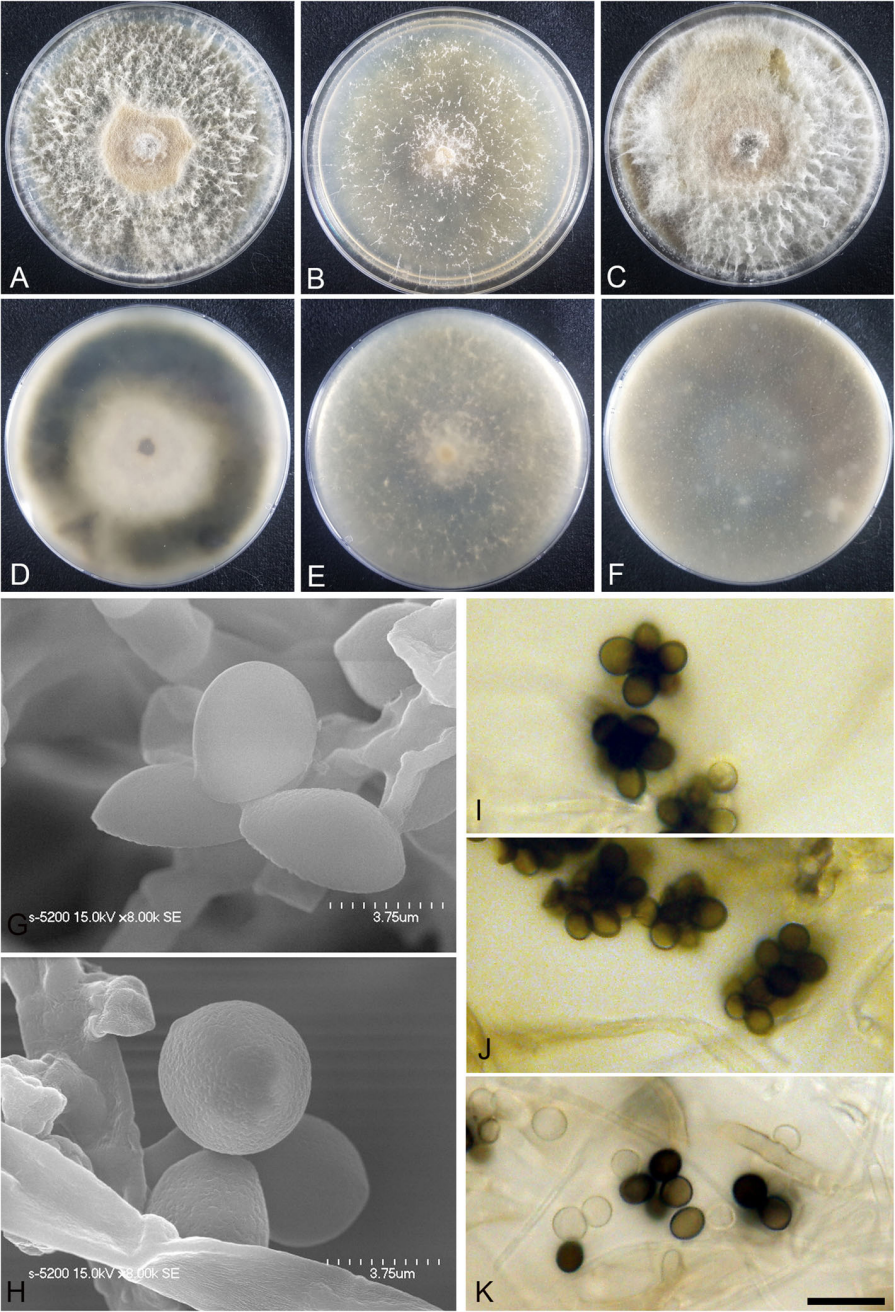
*Arthrinium pusillispermum* (KUC21321). **a-c**, Colonies on PDA (**a**), MEA (**b**), and OA (**c**) (top); **d-f**, colonies on PDA (**d**), MEA (**e**), and OA (**f**) (bottom); **g-h**, conidia under SEM; **i-k**, conidia attached to conidiogenous cells; scale bar = 10 μm

*Etymology*: ‘*pusillus*’, tiny and ‘*spermum*’ spores.

*Molecular diagnosis*: *Arthrinium pusillispermum* is distinguished from the phylogenetically most closely related species, *A. gutiae*, by unique single nucleotide polymorphisms in the three loci used in this study (Figs. 3S, 4S, 5S): ITS positions 43 (C), 260 (T), and 546 (T); TEF positions 1–17 (indel), 26–38 (indel), 43–46 (indel), 64–69 (indel), 76–82 (indel), 84–96 (indel), 112–115 (indel), 125–131 (indel), 137–141 (indel), 151–172 (indel), 173 (C), 174 (A), 175 (G), 178 (G), 180 (T), 192 (T), 193 (indel), 194 (G), 209 (A), 213 (indel), 228 (A), 230 (C), 243 (C), 251 (C), 252 (A), 256 (A), 260 (A), 261 (A), 264 (T), 268 (G), 269 (T), 273–276 (indel), 287–289 (indel), 293 (A), 294 (G), 308 (A), 310 (G), 313 (C), 314 (indel), 315 (C), 321 (T), 325 (indel), 327 (indel), 328 (A), 332 (indel), 337 (T), 356 (C), 358 (A), 360 (T), 364 (C), 374 (A), 395 (C), and 473 (T); TUB position 38 (C), 75 (T), 89 (G), 144 (A), and 498–506 (indel).

*Type*: **Korea:** Chungcheongnam-do, Taean-gun, 36°50′14.3″N, 126°11′04.7″E, isolated from Seaweed, 19 Mar. 2016, *S. Jang* (Herb. KCTC 46906 – holotype preserved in a metabolically inactive state; KUC21321 = NIBRFGC000501585 – ex-type culture).

*Description*: *Mycelium* consisting of smooth, hyaline, branched, septate, 1.5–4.5 μm diam. *Conidiogenous cells* aggregated in clusters on hyphae, hyaline, cylindrical. *Conidia* brown, smooth to granular, globose to subglobose in surface view, 4.0–6.0 (− 6.5) × (3.0–)3.5–5.0 (− 5.5) μm ($\overline{x}$ = 5.1 × 4.2 μm, *n* = 30); lenticular in side view, with equatorial slit, 3.5–4.5 μm wide ($\overline{x}$ = 4.1 μm, *n* = 30), elongated cell present.

*Culture*: PDA: colonies thick around centre, concentrically spreading with aerial mycelium, margin circular; mycelia white, pale yellow to grey; sporulation was not observed; greenish black (10GY 2.5/1) pigment diffused in medium; odour indistinct. MEA: colonies abundant, flat, concentrically spreading with sparse aerial mycelium, margin irregular; mycelia white to gray colored; sporulation was not observed; pigment absent in medium; odour indistinct. OA: colonies thick, concentrically spreading with aerial mycelium, margin irregular; mycelia white to pale brown and grey to dark grey; sporulation on hyphae around the centre after 2 weeks, spores black; greenish black (10Y 2.5/1) to very dark greenish grey (10Y 3/1) pigment diffused in medium; odour indistinct. *Colony diameters* (in mm after 120 h): 15 °C PDA 19–25, MEA 10–12, OA 11–12; 20 °C PDA 25–39, MEA 19–25, OA 22–24; 25 °C PDA 9–15, MEA 6–18, and OA 6–20.

*Additional material examined*: **Korea:** Chungcheongnam-do, Taean-gun, 36°50′14.3″N, 126°11′04.7″E, isolated from seaweed 19 Mar. 2016, *S. Jang* (KUC21357).

*Notes*: *Arthrinium pusillispermum* is closely related to *A. gutiae* (99.44% similarity in the ITS region, 88.52% in the TEF region, and 98.98% in the TUB region) (Figs. 1, 2). *Arthrinium pusillispermum* is distinguished from *A. gutiae* by the shape of the conidiogenous cells and the substrate: *A. pusillispermum* has cylindrical conidiogenous cells and was isolated from seaweed, whereas *A. gutiae* has lageniform conidiogenous cells and was isolated from the gut of grasshoppers (Crous et al. 2015). *Arthrinium pusillispermum* can be distinguished from the 22 non-sequenced species by its small conidia size (Table 2).

***Arthrinium sargassi*** S.L. Kwon, S. Jang & J.J. Kim, **sp. nov.**

MycoBank MB834598

(Fig. 9)

**Fig. 9:**
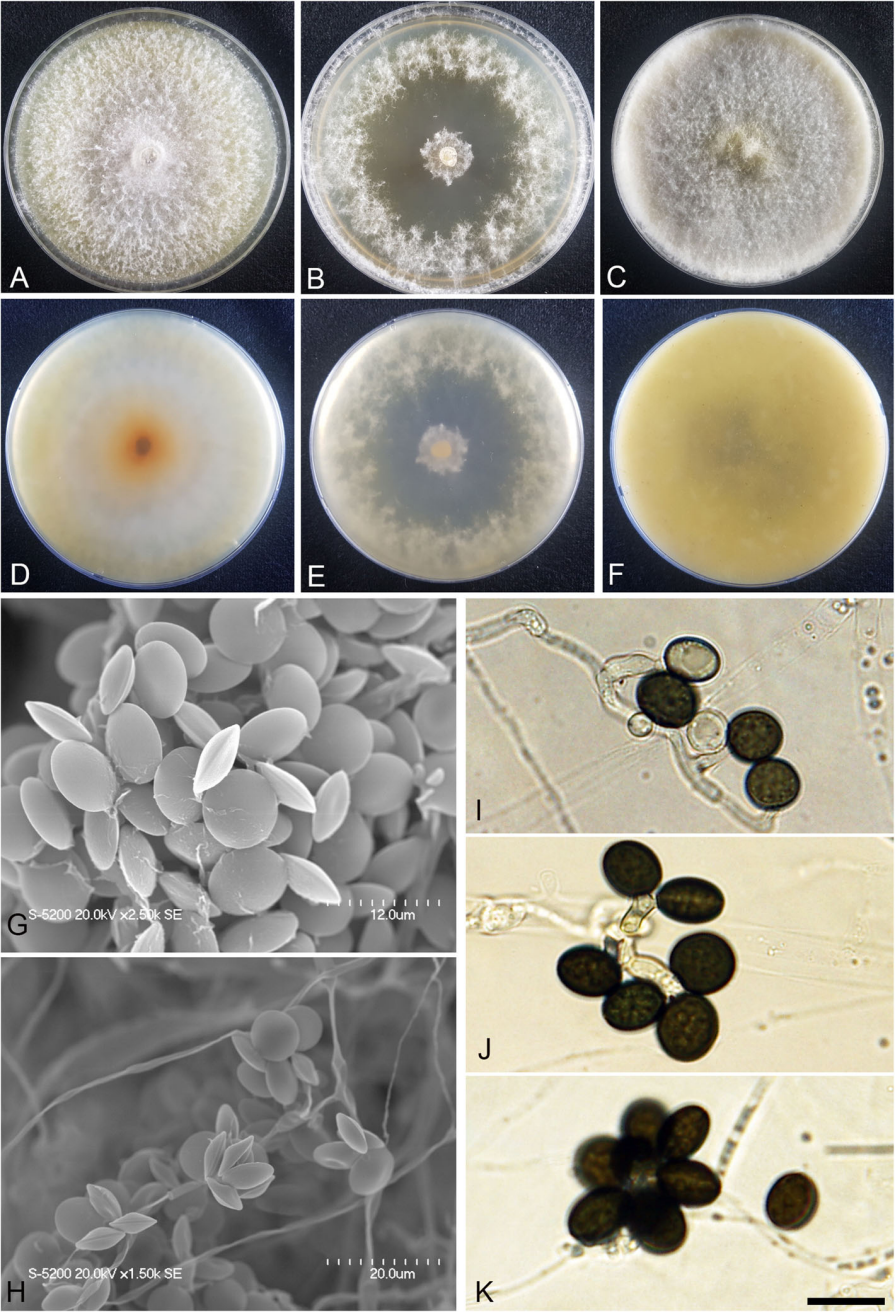
*Arthrinium sargassi* (KUC21232). **a-c**, Colonies on PDA (**a**), MEA (**b**), and OA (**c**) (top); **d-f**, colonies on PDA (**d**), MEA (**e**), and OA (**f**) (bottom); **g-h**, conidia under SEM; **i-k**, conidia attached to conidiogenous cells; scale bar = 10 μm

*Etymology*: ‘*sargassi’* refers to the genus name of *Sargassum* sp., the substrate of the type material.

*Molecular diagnosis*: *Arthrinium sargassi* is distinguished from the phylogenetically related species, *A. hydei*, by unique single nucleotide polymorphisms in the three loci used in this study (Figs. 3S, 4S, 5S): ITS positions 31 (C), 47 (indel), 91 (C), 95 (indel), 309 (T), and 644 (indel); TEF positions 15 (C), 27 (C), 30 (T), 37 (C), 46 (T), 47 (indel), 63 (indel), 64 (C), 66 (T), 67 (A), 92 (C), 93 (A), 95 (G), 140 (G), 152 (C), 153 (A), 155 (G), 160 (T), 193 (T), 222 (C), 224 (A), 225 (C), 253 (C), 254 (C), 262 (C), 265 (T), 293 (A), 328 (A), 336 (A), 358 (T), 367 (A), 371 (T), 374 (C), 376 (A), 386 (C), 392 (A), and 449 (C); TUB positions 10 (C), 18 (C), 22 (T), 23 (G), 30 (T), 45 (T), 47 (A), 50 (G), 52 (A), 69 (A), 70 (C), 80 (G), 106 (T), 133 (A), 145 (A), 225 (A), 230 (G), 380 (T), 416 (T), and 437 (T).

*Type*: **Korea:** Jeju-do, 33°23′39.2″N, 126°14′23.0″E, isolated from *Sargassum fulvellum*, 10 Jan. 2015, *S. Jang* (Herb. KCTC 46901 – holotype preserved in a metabolically inactive state; KUC21228 = NIBRFGC000501578 – ex-type culture).

*Description*: *Mycelium* consisting of smooth, hyaline, branched, septate, 2.0–5.0 μm diam. *Conidiogenous cells* aggregated in clusters on hyphae or solitary, at first hyaline, becoming pale brown, basauxic, polyblastic, sympodial, erect, cylindrical. *Conidia* brown, smooth to granular, globose to subglobose in surface view, (8.5–)9.5–11 (− 11.5) × (8.0–)8.5–10 (− 11) μm ($\overline{x}$ = 10.4 × 9.4 μm, *n* = 30); lenticular in side view, with equatorial slit, 5.5–7.5 μm wide ($\overline{x}$ = 6.5 μm, *n* = 30), elongated cell present.

*Culture*: PDA: colonies thick, flat, concentrically spreading with aerial mycelium, margin circular; mycelia white to grey, reverse sparsely pale yellow; sporulation on hyphae and in media after 2 weeks, randomly dense, spores black; yellow (10YR 8/8) pigment diffused in medium from centre, sometimes remaining as dark grey spots; odour indistinct. MEA: colonies slightly thick, flat, concentrically spreading with aerial mycelium, margin circular; mycelia white coloured; sporulation on hyphae and in media after 2 weeks, randomly dense, spores black; pigment absent, sometimes remaining dark grey spots in medium; odour indistinct. OA: colonies thick and dense, flat, concentrically spreading with aerial mycelium, margin circular; mycelia white, reverse usually yellow to green from the centre, sometimes becoming pinkish after 2 weeks; sporulation on hyphae, randomly dense after 2 weeks, spores black; yellow (2.5Y 7/8) pigment diffused in medium; odour indistinct. *Colony diameters* (in mm after 120 h): 15 °C PDA 10–12, MEA 15–23, OA 14–15; 20 °C PDA 21–26, MEA 20–27, OA 25–27; 25 °C PDA 29–32, MEA 26–28, and OA 30–34.

*Additional material examined*: **Korea:** Jeju-do, 33°23′39.2″N, 126°14′23.0″E, isolated from *Sargassum fulvellum*, 10 Jan. 2015, *S. Jang* (KUC21232, KUC21284, and KUC21287).

*Notes*: *Arthrinium sargassi* has morphological characteristics similar to those of other species in clade B. It can be distinguished from *A. aureum* (globose to ellipsoid conidia, 10–30 × 10–15 μm) and *A. hydei* (globose conidia, 17–19 μm diam) in the much smaller conidia, (8.5–)9.5–11 (− 11.5) × (8.0–)8.5–10 (− 11) μm ($\overline{x}$ = 10.4 × 9.4 μm, *n* = 30) (Calvo 1980; Crous & Groenewald 2013). *Arthrinium rasikravindrae* KUC21327 (34–39 mm in 5 d on PDA at 20 °C) and *A. marinum* (16–17 mm in 5 d on PDA at 20 °C) can be distinguished from *A. sargassi* (21–26 mm in 5 d on PDA at 20 °C) by their growth rate. Unfortunately, there are no data regarding the growth rate of *A. chinense*, but it can be clearly separated from *A. sargassi* based on the phylogenetic analysis (Figs. 1, 2). *Arthrinium sargassi* is morphologically similar to *A. sinensis*, a non-sequenced species. However, the shape of conidiogenous cell differs between them; lageniform in *A. sinensis* and cylindrical in *A. sargassi* (Table 2).

***Arthrinium taeanense*** S.L. Kwon, S. Jang & J.J. Kim, **sp. nov.**

MycoBank MB834599

(Fig. 10)

**Fig. 10:**
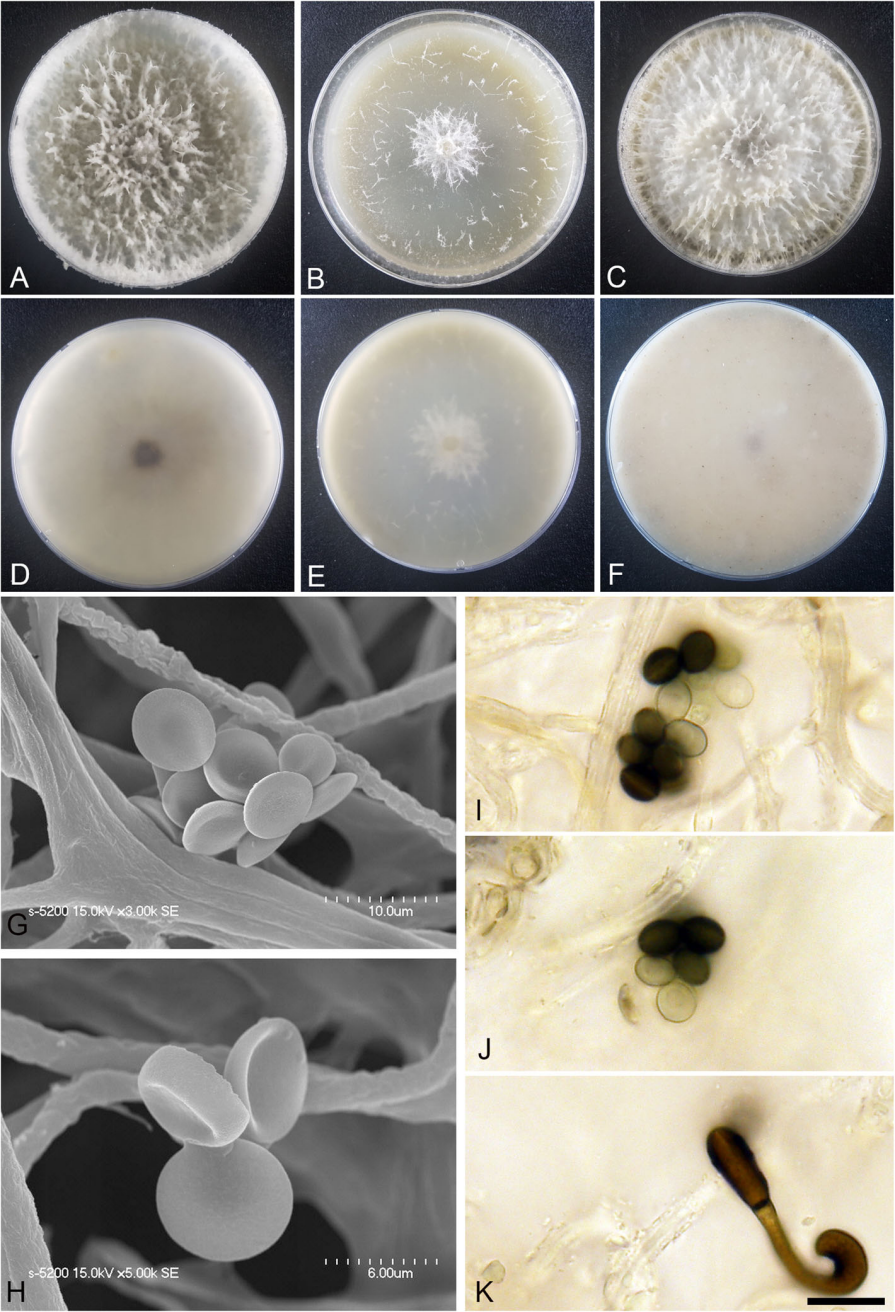
*Arthrinium taeanense* (KUC21322). A-C, Colonies on PDA (**a**), MEA (**b**), and OA (**c**) (top); **d-f**, colonies on PDA (**d**), MEA (**e**), and OA (**f**) (bottom); **g-h**, conidia under SEM; **i-k**, conidia attached to conidiogenous cells; scale bar = 10 μm

*Etymology*: ‘*taeanense’* refers to the type locality.

*Molecular diagnosis*: *Arthrinium taeanense* is distinguished from the phylogenetically most closely related species, *A. gutiae*, by unique single nucleotide polymorphisms in the three loci used in this study (Figs. 3S, 4S, 5S): ITS positions 22 (A), 32 (indel), 43 (G), 48 (C), 109 (indel), 113 (T), 121 (T), 129–146 (indel), 149–156 (indel), 189–192 (indel), 202–211 (indel), 213 (indel), 221 (T), 227–228 (indel), 248–250 (indel), 253 (C), 257 (T), 263 (A), 283 (G), 300 (T), 308 (C), 535 (C), 536 (G), 546 (T), 591 (A), 592 (T), and 593 (T); TEF positions 173 (T), 174 (C), 175 (A), 176 (C), 179 (C), 180 (T), 189 (G), 194 (G), 200 (indel), 209 (A), 213 (indel), 214 (C), 226 (A), 228 (A), 229 (A), 230 (C), 251 (C), 252 (T), 253 (T), 260 (A), 263 (C), 264 (T), 265 (A), 266 (T), 269 (T), 270 (T), 272 (G), 273–275 (indel), 278 (T), 280 (indel), 281 (A), 287 (G), 289 (C), 293 (A), 302 (indel), 304 (indel), 307 (G), 308 (G), 309 (indel), 310 (A), 313 (A), 314 (indel), 318 (G), 334 (G), 337 (T), 356 (A), 357 (G), 358 (A), 371 (T), 374 (A), 375 (G), 376 (G), 378 (C), 395 (C), 404 (C), 467 (T), and 600 (C); TUB positions 2 (T), 3 (C), 7 (C), 10 (C), 11–12 (indel), 16 (G), 17 (T), 19 (A), 20 (C), 21 (A), 22 (T), 23 (C), 25 (C), 26 (G), 28 (G), 29 (A), 33 (C), 34 (C), 35 (T), 36 (C), 38 (C), 41 (T), 44 (A), 46 (G), 53 (A), 54 (T), 68 (T), 69 (C), 71 (A), 72 (A), 73 (T), 74 (A), 75 (T), 78 (T), 80 (G), 81 (C), 85 (G), 87 (G), 89 (G), 95 (C), 108 (G), 111 (G), 114 (A), 129 (T), 138 (C), 140 (T), 143 (T), 146 (T), 158 (C), 170 (C), 176 (C), 184 (A), 198 (C), 205 (A), 207 (C), 211–212 (indel), 214–216 (indel), 231 (G), 308 (C), 309 (C), 312 (C), 313 (T), 319 (T), 324 (C), 326 (G), 327 (C), 328 (C), 329 (T), 344 (T), 347 (T), 353 (C), 392 (A), 395 (T), 410 (C), 413 (G), 416 (C), 425 (C), 428 (T), 434 (C), 437 (G), 455 (T), 476 (T), 479 (C), and 485 (C).

*Type*: **Korea:** Chungcheongnam-do, Taean-gun, 36°50′14.3″N, 126°11′04.7″E, isolated from Seaweed, 19 Mar. 2016, *S. Jang* (Herb. KCTC 46910 – holotype preserved in a metabolically inactive state; KUC21322 = NIBRFGC000501589 – ex-type culture).

*Description*: *Mycelium* consisting of smooth, hyaline, branched, septate, 2.0–4.5 μm diam. *Conidiogenous cells* aggregated in clusters on hyphae, hyaline, cylindrical. *Conidia* brown, smooth to granular, globose to elongate ellipsoid in surface view, (5.0–)5.5–6.5 (− 7.0) × 4.0–5.5 (− 6.0) μm ($\overline{x}$ = 6 × 4.7 μm, *n* = 30); lenticular in side view, with an equatorial slit, 4.0–5.0 μm wide ($\overline{x}$ = 4.7 μm, *n* = 30), elongated cell observed.

*Culture*: PDA, colonies thick, concentrically spreading with aerial mycelium, margin circular; mycelia white to yellow, gray and partially pale orange colored; sporulation was not observed; pale yellow (5Y 8/3) pigment to yellow (2.5Y 8/8) pigment diffused in media after 2 weeks; odour indistinct. MEA, colonies thick, flat, concentrically spreading with aerial mycelium, margin circular; mycelia white to yellowish gray colored; sporulation was not observed; pigment absent in medium; odour indistinct. OA, colonies very thick, concentrically spreading with aerial mycelium, margin circular; mycelia white to yellow and orange to brown colored; sporulation was not observed; yellowish brown (10YR 5/8) pigment diffused in media after 2 weeks; odour indistinct. *Colony diameters* (in mm after 120 h): 15 °C PDA 7–15, MEA 10–20, OA 10–11; 20 °C PDA 28–36, MEA 24–32, OA 21–24; 25 °C PDA 36–39, MEA 34–35, and OA 39–41.

*Additional material examined*: **Korea:** Chungcheongnam-do, Taean-gun, 36°50′14.3″N, 126°11′04.7″E, isolated from seaweed, 19 Mar. 2016, *S. Jang* (KUC21358, KUC21359).

*Notes*: *Arthrinium taeanense* is most closely related to *A. pusillispermum* (95.30% similarity in the ITS region, 80.84% in the TEF region, and 79.30% in the TUB region) and *A. gutiae* (95.30% similarity in the ITS region, 85.19% in the TEF region, and 78.3% in the TUB region) (Fig. 1). There were no noticeable morphological characters that helped separate these species, but the long stem branches clearly indicate that they represent different, phylogenetically well-separated taxa. *Arthrinium taeanense* can be distinguished from the 22 non-sequenced species by its small conidia size (Table 2).

## DISCUSSION

A total of 14 *Arthrinium* species associated with marine environments in Korea was identified based on morphological and molecular phylogenetic analyses. Five species, *A. arundinis*, *A. marii*, *A. rasikravindrae*, *A. sacchari*, and *A. saccharicola,* had already been reported from marine environments (Hong et al. 2015; Park et al. 2018), whereas *A. piptatheri* was reported here for the first time from this habitat. The newly recognized taxa represented six species isolated from macroalgae (*A. agari, A. fermenti, A. marinum, A. pusillispermum, A. sargassi,* and *A. taeanense*) and two extracted from the egg masses of sailfin sandfish (*A. arctoscopi* and *A. koreanum*). To date, the majority of the described *Arthrinium* species have been isolated from various terrestrial habitats (Tsukamoto et al. 2006; Kim et al. 2011; Crous & Groenewald 2013), whereas only eight *Arthrinium* species have been reported from marine environments: *A. algicola*, *A. arundinis*, *A. hispanicum*, *A. marii*, *A. phaeospermum*, *A. rasikravindrae*, *A. sacchari*, and *A. saccharicola* (Miao et al. 2006; Jones et al. 2009; Crous & Groenewald 2013; Hong et al. 2015; Larrondo 1992; Li et al. 2017; Park et al. 2018; Pintos et al. 2019).

As mentioned, conidial shape, conidiophores, and presence or absence of sterile cells and setae were previously used for the infrageneric classification and delimitation of species (Schmidt & Kunze 1817; Hughes 1953; Minter 1985). However, because these microscopic features often overlap between taxa, it is difficult to solely rely on them to distinguish species. Therefore, the combined use of molecular and morphological characters, in combination with the physiological features of the cultures, is required to identify species in *Arthrinium*. For example, the newly recognized species, *A. marinum, A. pusillispermum*, and *A. taeanense*, cannot be distinguished from their close relatives based on morphology alone; however, the three species could be distinguished by differences in their growth rate and by the molecular data.

*Arthrinium* species can be divided into two groups based on conidial shape: one group with an irregular conidial shape, similar to a cashew-nut (*A. kamischaticum*) or a polygon (*A. puccinioides*), and the other with globose to ellipsoid conidia (Singh et al. 2013). All *Arthrinium* species in this study produced globose to subglobose or globose to ellipsoid conidia. This corresponds to the conidial shape of other *Arthrinium* species derived from marine environments (Larrondo 1992; Crous and Groenewald 2013; Singh et al. 2013). Among the species with ellipsoid conidia, those from marine environments generally have more elongated conidia than those from terrestrial environments (Table 2). There are a number of *Arthrinium* species described only from their sexual morph (e.g., *A. balearicum*, *A. garethjonesii*, *A. longistromum*, *A. neosubglobosa*, *A. subglobosa*) (Senanayake et al. 2015; Dai et al. 2016; Dai et al. 2017; Pintos et al. 2019). Unfortunately, no sexual morph is known in any of the marine species. This further increases the difficulty of identifying *Arthrinium* species through morphological features alone.

DNA sequencing data available for *Arthrinium* species has been steadily increasing in recent years (Crous and Groenewald 2013; Wang et al. 2018; Pintos et al. 2019). Currently 84 species of *Arthrinium* are recognized; of these, sequence information on the ITS is available for 62 species, TUB for 51, and TEF for 45 species. This has contributed to an increase in newly recognized species and aids in their accurate and rapid identification (Wang et al. 2018; Pintos et al. 2019). ITS by itself is limited in its ability to identify species within *Arthrinium*. The use of TUB, TEF, and multigene sequence data (ITS, TUB, and TEF) has increased the accurate identification and phylogenetic relationships in *Arthrinium*. This study generated 67 sequence datasets for three gene regions (ITS, TUB, and TEF), which will also contribute to furthering the study of the genus *Arthrinium*.

According to our previous studies on marine *Arthrinium* species, the 14 identified in this study can be expected to have high biological activity. However, it is not clear whether they are active in the actual marine environment and what the ecological role of *Arthrinium* species is. We expect to better understand their role in the environment through various studies of *Arthrinium* species in the future, including the discovery of further novel species and an exploration of their biological properties.

## CONCLUSIONS

Our study underlines the notion that the diversity of *Arthrinium* species is still poorly known. More than half of the *Arthrinium* species isolated from a limited marine environment resulted to be new to science. According to our results, many more novel taxa are to be expected from marine environments around the world. Further studies in other environments are needed to assess the distribution of these species. Our results also show that a polyphasic approach to the taxonomy of *Arthrinium*, integrating molecular phylogeny of ITS and protein-coding markers, conidial features and culture characteristics are the most reliable approach to delimit and recognize species in this genus.

## Supplementary Information

**Additional file 1: Fig. S1.** ML tree based on the TEF region. The numbers at the nodes indicate ML bootstrap support (BS) > 75% and Bayesian posterior probabilities (PP) > 0.75 as BS/PP. The thickened branches indicate support greater than 85% for BS and 0.95 for PP. A hyphen (‘-‘) indicates values of BS < 70% or PP < 0.75. Ex-holotype strains are indicated with asterisks (‘*’). The fungal cultures examined in this study are shown in bold. Red boxes indicate the novel species. The numbers in the brackets indicate strain number. The scale bar indicates the nucleotide substitutions per position. **Fig. S2.** ML tree based on the TUB region. The numbers at the nodes indicate ML bootstrap support (BS) > 75% and Bayesian posterior probabilities (PP) > 0.75 as BS/PP. The thickened branches indicate support greater than 85% for BS and 0.95 for PP. A hyphen (‘-‘) indicates values of BS < 70% or PP < 0.75. Ex-holotype strains are indicated with asterisks (‘*’). The fungal cultures examined in this study are shown in bold. Red boxes indicate the novel species. The numbers in the brackets indicate strain number. The scale bar indicates the nucleotide substitutions per position. **Fig. S3.** Sequence alignments of ITS regions of eight novel *Arthrinium*. **Fig. S4.** Sequence alignments of TEF regions of eight novel *Arthrinium.* **Fig. S5.** Sequence alignments of TUB regions of eight novel *Arthrinium.*

**Additional file 2: Table S1.** Sequence information of Arthrinium species. Newly established species in this study are shown in bold.

## Data Availability

All data generated or analyzed during this study are included in this published article.

## Abbreviations

BI: Bayesian tree inference
bp: base pair
BS: Bootstrap support
diam: diameter
DNA: Deoxyribonucleic acid
Herb: Herbarium
ITS: Internal transcribed spacer
KCTC: Korean Collection for Type Culture
KUC: Korea University Fungus Collection
MEA: Malt extract agar
ML: Maximum likelihood
OA: oatmeal agar
PCR: Polymerase chain reaction
PDA: Potato dextrose agar
PP: Posterior probabilities
ROS: Reactive oxygen species
SEM: Scanning electron microscope
SFC: Seoul National University Fungus Collection
TEF: Translation elongation factor 1-alpha
TUB: *β*-tubulin

## References

[CR1] Calvo A (1980). Arthrinium aureum sp. nov. from Spain. Transactions of the British Mycological Society.

[CR2] Carbone I, Kohn LM (1999). A method for designing primer sets for speciation studies in filamentous ascomycetes. Mycologia.

[CR3] Cooke WB (1954). The genus Arthrinium. Mycologia.

[CR4] Crous PW, Groenewald JZ (2013). A phylogenetic re-evaluation of Arthrinium. IMA Fungus.

[CR5] Crous PW, Verkley GJ, Groenewald JZ, Samson R (2009). Fungal biodiversity.

[CR6] Crous PW, Wingfield MJ, Le Roux JJ, Richardson DM, Strasberg D (2015). Fungal planet description sheets: 371–399. Persoonia.

[CR7] Dai D, Jiang H, Tang L, Bhat D (2016). Two new species of Arthrinium (Apiosporaceae, Xylariales) associated with bamboo from Yunnan, China. Mycosphere.

[CR8] Dai DQ, Phookamsak R, Wijayawardene NN, Li WJ, Bhat DJ, Xu JC, Taylor JE, Hyde KD, Chukeatirote E (2017). Bambusicolous fungi. Fungal Diversity.

[CR9] Darriba D, Taboada GL, Doallo R, Posada D (2012). jModelTest 2: more models, new heuristics and parallel computing. Nature Methods.

[CR10] Elissawy AM, Ebada SS, Ashour ML, Özkaya FC, Ebrahim W, Singab ANB, Proksch P (2017). Spiroarthrinols a and B, two novel meroterpenoids isolated from the sponge-derived fungus *Arthrinium* sp. Phytochemistry Letters.

[CR11] Ellis MB (1972). Dematiaceous hyphomycetes: XI. Mycological Papers.

[CR12] Flewelling AJ, Currie J, Gray CA, Johnson JA (2015). Endophytes from marine macroalgae: promising sources of novel natural products. Current Science.

[CR13] Fungi of Great Britain and Ireland (FGBI) (n.d.). http://fungi.myspecies.info/all-fungi/arthrinium-morthieri. Accessed 13 Jan 2021

[CR14] Gardes M, Bruns TD (1993). ITS primers with enhanced specificity for basidiomycetes-application to the identification of mycorrhizae and rusts. Molecular Ecology.

[CR15] Glass NL, Donaldson GC (1995). Development of primer sets designed for use with the PCR to amplify conserved genes from filamentous ascomycetes. Applied and Environmental Microbiology.

[CR16] Harvard University Herbaria & Libraries (HUH) (n.d.). https://huh.harvard.edu/. Accessed 13 Jan 2021

[CR17] Heo YM, Kim K, Ryu SM, Kwon SL, Park MY, Kang JE, Hong JH, Lim YW, Kim C, Kim BS, Lee D, Kim JJ (2018). Diversity and ecology of marine Algicolous *Arthrinium* species as a source of bioactive natural products. Marine Drugs.

[CR18] Hong J-H, Jang S, Heo YM, Min M, Lee H, Lee Y, Lee H, Kim JJ (2015). Investigation of marine-derived fungal diversity and their exploitable biological activities. Marine Drugs.

[CR19] Hughes SJ (1953). Conidiohores, conidia, and classification. Canadian Journal of Botany.

[CR20] Hyde KD, Frohlich J, Taylor JE (1998). Fungi from palms. XXXVI. Reflections on unitunicate ascomycetes with apiospores. Sydowia.

[CR21] Hyde KD, Hongsanan S, Jeewon R, Bhat DJ, McKenzie EHC (2016). Fungal diversity notes 367–490: taxonomic and phylogenetic contributions to fungal taxa. Fungal Diversity.

[CR22] Hyde KD, Norphanphoun C, Maharachchikumbura SSN, Bhat DJ, Jones EBG (2020). Refined families of *Sordariomycetes*. Mycosphere.

[CR23] Jiang HB, Hyde KD, Doilom M, Karunarathna SC, Xu JC (2019). Arthrinium setostromum (Apiosporaceae, Xylariales), a novel species associated with dead bamboo from Yunnan, China. Asian Journal of Mycology.

[CR24] Jiang N, Li J, Tian CM (2018). *Arthrinium* species associated with bamboo and reed plants in China. Fungal System Evolution.

[CR25] Jiang N, Liang YM, Tian CM (2020). A novel bambusicolous fungus from China, *Arthrinium chinense* (Xylariales). Sydowia.

[CR26] Joint Publications Research Service Arlington (JPRSA) VA (1977). People's Republic of China Scientific Abstracts.

[CR27] Jones EBG, Sakayaroj J, Suetrong S, Somrithipol S, Pang KL (2009). Classification of marine *Ascomycota*, anamorphic taxa and *Basidiomycota*. Fungal Diversity.

[CR28] Katoh K, Standley DM (2013). MAFFT multiple sequence alignment software version 7: improvements in performance and usability. Molecular Biology and Evolution.

[CR29] Kim J-J, Lee S-S, Ra J-B, Lee H, Huh N, Kim GH (2011). Fungi associated with bamboo and their decay capabilities. Holzforschung.

[CR30] Kumar S, Stecher G, Tamura K (2016). MEGA7: molecular evolutionary genetics analysis version 7.0 for bigger datasets. Molecular Biology and Evolution.

[CR31] Larrondo J (1992). New contributions to the study of the genus *Arthrinium*. Mycologia.

[CR32] Larrondo J, Calvo MA (1990). Two new species of *Arthrinium* from Spain. Mycologia.

[CR33] Li Y, Wang J, He W, Lin X, Zhou X, Liu Y (2017). One strain-many compounds method for production of polyketide metabolites using the sponge-derived fungus *Arthrinium arundinis* ZSDS1-F3. Chemistry of Natural Compounds.

[CR34] Luo Z-L, Hyde KD, Liu J-K, Maharachchikumbura SSN, Jeewon R (2019). Freshwater *Sordariomycetes*. Fungal Diversity.

[CR35] Miao L, Kwong TF, Qian P-Y (2006). Effect of culture conditions on mycelial growth, antibacterial activity, and metabolite profiles of the marine-derived fungus *Arthrinium* c.f. *saccharicola*. Applied Microbiology and Biotechnology.

[CR36] Miller MA, Pfeiffer W, Schwartz T (2010). Creating the CIPRES science gateway for inference of large phylogenetic trees. 2010 gateway computing environments workshop (GCE): 1-8.

[CR37] Minter D (1985). A re-appraisal of the relationships between *Arthrinium* and other hyphomycetes. Plant Science.

[CR38] Minter DW, Cannon PF (2018). IMI descriptions of Fungi and Bacteria 216, sheets 2151–2160. CABI Wallingford, UK.

[CR39] Mopper K, Kieber DJ (2000). Marine photochemistry and its impact on carbon cyclin. The effects of UV radiation in the marine environment.

[CR40] Munsell Color (2009). Munsell soil-color charts with genuine Munsell color chips. Munsell Color, Grand Rapids, MI, USA.

[CR41] O’Donnell K, Kistler HC, Cigelnik E, Ploetz RC (1998). Multiple evolutionary origins of the fungus causing Panama disease of banana: concordant evidence from nuclear and mitochondrial gene genealogies. Proceedings of the National Academy of Sciences of the United States of America.

[CR42] O'Donnell K, Cigelnik E (1997). Two divergent intragenomic rDNA ITS2 types within a monophyletic lineage of the fungus *Fusarium* are nonorthologous. Molecular Phylogenetics and Evolution.

[CR43] Park MS, Oh S-Y, Lee S, Eimes JA, Lim YW (2018). Fungal diversity and enzyme activity associated with sailfin sandfish egg masses in Korea. Fungal Ecology.

[CR44] Pintos Á, Alvarado P, Planas J, Jarling R (2019). Six new species of *Arthrinium* from Europe and notes about *A. caricicola* and other species found in *Carex* spp. hosts. MycoKeys.

[CR45] Pollack FG, Benjamin CR (2020). *Arthrinium japonicum* and notes on *Arthrinium kamtschaticum*. Mycologia.

[CR46] Rabenhorst L, Lindau G (1907). Dr. L. Rabenhorst's Kryptogamen-Flora von Deutschland, Oesterreich und der Schweiz: Hyphomycetes (erste Hälfte) Mucedinaceae, Dematiaceae (Phaeosporae und Phaeodidymae) vol 1 part 8. E. Kummer, Leipzig, Germany.

[CR47] Rambelli A, Venturella G, Ciccarone C (2008). More dematiaceous hyphomycetes from *Pantelleria mediterranea* maquis litter. Flora Mediterranea.

[CR48] Ronquist F, Teslenko M, Van Der Mark P, Ayres DL, Darling A (2012). MrBayes 3.2: efficient Bayesian phylogenetic inference and model choice across a large model space. Systematic Biology.

[CR49] Schmidt J, Kunze G (1817). Mykologische Hefte. 1. Vossische Buchhandlung, Leipzig.

[CR50] Senanayake IC, Maharachchikumbura SS, Hyde KD, Bhat JD, Jones EG (2015). Towards unraveling relationships in *Xylariomycetidae* (*Sordariomycetes*). Fungal Diversity.

[CR51] Sharma R, Kulkarni G, Sonawane MS, Shouche YS (2014). A new endophytic species of *Arthrinium* (*Apiosporaceae*) from *Jatropha podagrica*. Mycoscience.

[CR52] Singh SM, Yadav LS, Singh PN, Hepat R, Sharma R, Singh SK (2013). *Arthrinium rasikravindrii* sp. nov. from Svalbard, Norway. Mycotaxon.

[CR53] Stamatakis A (2006). RAxML-VI-HPC: maximum likelihood-based phylogenetic analyses with thousands of taxa and mixed models. Bioinformatics.

[CR54] Sukova M (2004). Fungi on Juncus trifidus in the Czech Republic. I. Czech Mycology.

[CR55] Suryanarayanan T (2012). Fungal endosymbionts of seaweeds. Biology of Marine Fungi. Springer.

[CR56] Tsukamoto S, Yoshida T, Hosono H, Ohta T, Yokosawa H (2006). Hexylitaconic acid: a new inhibitor of p53–HDM2 interaction isolated from a marine-derived fungus, *Arthrinium* sp. Bioorganic & Medicinal Chemistry Letters.

[CR57] Wang M, Liu F, Crous P, Cai L (2017). Phylogenetic reassessment of *Nigrospora*: ubiquitous endophytes, plant and human pathogens. Persoonia.

[CR58] Wang M, Tan X-M, Liu F, Cai L (2018). Eight new *Arthrinium* species from China. MycoKeys.

[CR59] Wei M-Y, Xu R-F, Du S-Y, Wang C-Y, Xu T-Y (2016). A new griseofulvin derivative from the marine-derived *Arthrinium* sp. fungus and its biological activity. Chemistry of Natural Compounds.

[CR60] White TJ, Bruns T, Lee S, Taylor J (1990). Amplification and direct sequencing of fungal ribosomal RNA genes for phylogenetics, vol 18. PCR protocols: a guide to methods and applications, vol 1.

[CR61] Yan H, Jiang N, Liang L-Y, Yang Q, Tian CM (2019). *Arthrinium trachycarpum* sp. nov. from *Trachycarpus fortunei* in China. Phytotaxa.

[CR62] Yang C-L, Xu X-L, Dong W, Wanasinghe DN, Liu Y-G (2019). Introducing *Arthrinium phyllostachium* sp. nov. (*Apiosporaceae*, *Xylariales*) on *Phyllostachys heteroclada* from Sichuan Province, China. Phytotaxa.

[CR63] Zhao YZ, Zhang ZF, Cai L, Peng WJ, Liu F (2018). Four new filamentous fungal species from newly-collected and hive-stored bee pollen. Mycosphere.

