# Correction to: The genus Arthrinium (Ascomycota, Sordariomycetes, Apiosporaceae) from marine habitats from Korea, with eight new species

**DOI:** 10.1186/s43008-022-00093-3

**Published:** 2022-05-16

**Authors:** Michael Sun Lul Kwon, Myung Soo Park, Seokyoon Jang, Young Min Lee, Young Mok Heo, Joo-Hyun Hong, Hanbyul Lee, Yeongseon Jang, Ji-Hyun Park, Changmu Kim, Gyu-Hyeok Kim, Young Woon Lim, Jae-Jin Kim

**Affiliations:** 1 Division of Environmental Science & Ecological Engineering, College of Life Science & Biotechnology, Korea University, Seoul, 02841 South Korea; 2 School of Biological Sciences and Institute of Microbiology, Seoul National University, Seoul, 08826 South Korea; 3 Division of Wood Chemistry and Microbiology, National Institute of Forest Science, Seoul, 02455 South Korea; 4 Microorganism Resources Division, National Institute of Biological Resources, Incheon, 22689 South Korea

## Correction to: IMA Fungus (2021) 12:1 10.1186/s43008-021-00065-z

Following the publication of the original article (Kwon et al. 2021), we were notified that Figs. 6 and 7 have been swapped. Figure 6 actually depicts the Arthrinium marinum, while Fig. 7 shows Arthrinium koreanum.

The original article has been corrected.
