# Supplementary material for: The genus Arthrinium (Ascomycota, Sordariomycetes, Apiosporaceae) from marine habitats from Korea, with eight new species
Source: IMA Fungus. 2021 Jun 1;12:13. doi: 10.1186/s43008-021-00065-z (PMC8168325; doi:10.1186/s43008-021-00065-z)
Supplement: Supplementary file 1 — Additional file 1: Fig. S1. ML tree based on the TEF region. The numbers at the nodes indicate ML bootstrap support (BS) > 75% and Bayesian posterior probabilities (PP) > 0.75 as BS/PP. The thickened branches indicate support greater than 85% for BS and 0.95 for PP. A hyphen (‘-‘) indicates values of BS < 70% or PP < 0.75. Ex-holotype strains are indicated with asterisks (‘*’). The fungal cultures examined in this study are shown in bold. Red boxes indicate the novel species. The numbers in the brackets indicate strain number. The scale bar indicates the nucleotide substitutions per position. Fig. S2. ML tree based on the TUB region. The numbers at the nodes indicate ML bootstrap support (BS) > 75% and Bayesian posterior probabilities (PP) > 0.75 as BS/PP. The thickened branches indicate support greater than 85% for BS and 0.95 for PP. A hyphen (‘-‘) indicates values of BS < 70% or PP < 0.75. Ex-holotype strains are indicated with asterisks (‘*’). The fungal cultures examined in this study are shown in bold. Red boxes indicate the novel species. The numbers in the brackets indicate strain number. The scale bar indicates the nucleotide substitutions per position. Fig. S3. Sequence alignments of ITS regions of eight novel Arthrinium. Fig. S4. Sequence alignments of TEF regions of eight novel Arthrinium. Fig. S5. Sequence alignments of TUB regions of eight novel Arthrinium. [file 43008_2021_65_MOESM1_ESM.zip › supplementary (Fig legend).docx]

**Fig. 1S** ML tree based on the TEF region. The numbers at the nodes indicate ML bootstrap support (BS) > 75% and Bayesian posterior probabilities (PP) > 0.75 as BS/PP. The thickened branches indicate support greater than 85% for BS and 0.95 for PP. A hyphen (‘-‘) indicates values of BS < 70% or PP < 0.75. Ex-holotype strains are indicated with asterisks (‘*’). The fungal cultures examined in this study are shown in bold. Red boxes indicate the novel species. The numbers in the brackets indicate strain number. The scale bar indicates the nucleotide substitutions per position.

**Fig. 2S** ML tree based on the TUB region. The numbers at the nodes indicate ML bootstrap support (BS) > 75% and Bayesian posterior probabilities (PP) > 0.75 as BS/PP. The thickened branches indicate support greater than 85% for BS and 0.95 for PP. A hyphen (‘-‘) indicates values of BS < 70% or PP < 0.75. Ex-holotype strains are indicated with asterisks (‘*’). The fungal cultures examined in this study are shown in bold. Red boxes indicate the novel species. The numbers in the brackets indicate strain number. The scale bar indicates the nucleotide substitutions per position.

**Fig. 3S** Sequence alignments of ITS regions of eight novel *Arthrinium*

**Fig. 4S** Sequence alignments of TEF regions of eight novel *Arthrinium*

**Fig. 5S** Sequence alignments of TUB regions of eight novel *Arthrinium*
