# Supplementary material for: The genus Arthrinium (Ascomycota, Sordariomycetes, Apiosporaceae) from marine habitats from Korea, with eight new species
Source: IMA Fungus. 2021 Jun 1;12:13. doi: 10.1186/s43008-021-00065-z (PMC8168325; doi:10.1186/s43008-021-00065-z)
Supplement: Supplementary file 2 — Additional file 2: Table S1. Sequence information of Arthrinium species. Newly established species in this study are shown in bold. [file 43008_2021_65_MOESM2_ESM.docx]

**Table 1S** Sequence information of *Arthrinium* species. Newly established species in this study are shown in bold.

| **Species** | **Sequence** | | |
| --- | --- | --- | --- |
|  | **ITS** | **TUB** | **TEF** |
| ***A. agari*** | **MH498520** | **MH498478** | **MH544663** |
| *A. algicola* | - | - | - |
| *A. aquaticum* | MK828608 | - | - |
| ***A. arctoscopi*** | **MH498529** | **MH498487** | **MN868918** |
| *A. arundinis* | KF144885 | KF144975 | KF145017 |
| *A. aureum* | AB220251 | KF144981 | KF145023 |
| *A. austriacum* | - | - | - |
| *A. balearicum* | MK014869 | MK017975 | MK017946 |
| *A. bambusae* | KY494718 | KY705186 | KY806204 |
| *A. camelliae-sinensis* | KY494704 | KY705173 | KY705103 |
| *A. caricicola* | MK014871 | MK017977 | MK017948 |
| *A. carinatum* | - | - | - |
| *A. chinense* | MK819291 | MK818547 | MK818545 |
| *A. cuspidatum* | - | - | - |
| *A. descalsii* | MK014870 | MK017976 | MK017947 |
| *A. dichotomanthi* | KY494697 | KY705167 | KY705096 |
| *A. esporlense* | MK014878 | MK017983 | MK017954 |
| *A. euphorbiae* | AB220241 | AB220288 | - |
| ***A. fermenti*** | **MF615226** | **MF615231** | **MH544667** |
| *A. fuckelii* | - | - | - |
| *A. gaoyouense* | MH197124 | MH236789 | MH236793 |
| *A. garethjonesii* | KY356086 | - | - |
| *A. globosum* | - | - | - |
| *A. guizhouense* | KY494708 | KY705177 | KY705107 |
| *A. gutiae* | KR011352 | KR011350 | KR011351 |
| *A. gutta* | - | - | - |
| *A. hispanicum* | AB220242 | AB220289 | - |
| *A. hydei* | KF144890 | KF144982 | KF145024 |
| *A. hyphopodii* | NR_154699 | - | - |
| *A. hysterinum* | MK014876 | MK017981 | MK017952 |
| *A. ibericum* | MK014879 | MK017984 | MK017955 |
| *A. italicum* | MK014880 | MK017985 | MK017956 |
| *A. japonicum* | AB220262 | AB220309 | - |
| *A. jatrophae* | JQ246355 | - | - |
| *A. jiangxiense* | KY494686 | KY806201 | KY705085 |
| *A. kamtschaticum* | - | - | - |
| *A. kogelbergense* | KF144891 | KF144983 | KF145025 |
| ***A. koreanum*** | **MH498524** | **MH498482** | **MH544664** |
| *A. leucospermum* | - | - | - |
| *A. lobatum* | - | - | - |
| *A. locutum-pollinis* | MF939595 | MF939622 | MF939616 |
| *A. longistromum* | KU940141 | - | - |
| *A. luzulae* | - | - | - |
| *A. macrosporum* | - | - | - |
| *A. malaysianum* | KF144897 | KF144989 | KF145031 |
| *A. marii* | KF144899 | KF144991 | KF145033 |
| ***A. marinum*** | **MH498538** | **MH498496** | **MH544669** |
| *A. mediterranei* | AB220243 | AB220290 | - |
| *A. minus* | MK014872 | MK017978 | MK017949 |
| *A. morthieri* | - | - | - |
| *A. muelleri* | - | - | - |
| *A. mytilomorphum* | KY494685 | - | - |
| *A. naviculare* | - | - | - |
| *A. neogarethjonesii* | NR_171943 | - | - |
| *A. neosubglobosa* | NR_154737 | - | - |
| *A. obovatum* | KY494696 | KY705166 | KY705095 |
| *A. ovatum* | KF144903 | KF144995 | KF145037 |
| *A. paraphaeospermum* | MT040110 | MT040152 | MT040131 |
| *A. phaeospermum* | KF144905 | KF144997 | KF145039 |
| *A. phragmitis* | KF144909 | KF145001 | KF145043 |
| *A. phyllostachydis* | MK351842 | MK291949 | MK340918 |
| ***A. piptatheri*** | **KT207736** | **KT207636** | **MH544672** |
| *A. pseudoparenchymaticum* | KY494743 | KY705211 | KY705139 |
| *A. pseudosinense* | KF144910 | **MN868936** | KF145044 |
| *A. pseudospegazzinii* | KF144911 | KF145002 | KF145045 |
| *A. pterospermum* | KF144913 | KF145004 | KF145046 |
| *A. puccinioides* | MK014894 | MK017998 | MK017970 |
| ***A. pusillispermum*** | **MH498533** | **MH498491** | **MN868930** |
| *A. qinlingense* | MH197120 | MH236791 | MH236795 |
| ***A. rasikravindrae*** | **MH498540** | **MH498498** | **MN868932** |
| *A. sacchari* | KF144916 | KF145005 | KF145047 |
| *A. saccharicola* | KF144921 | KF145011 | KF145053 |
| ***A. sargassi*** | **KT207746** | **KT207644** | **MH544677** |
| *A. scriptum* | - | - | - |
| *A. serenense* | AB220250 | AB220297 | - |
| *A. setostromum* | MN528012 | - | MN527357 |
| *A. sinensis* | - | - | - |
| *A. spegazzinii* | - | - | - |
| *A. sphaerospermum* | - | - | - |
| *A. sporophlaeum* | - | - | - |
| *A. sporophleoides* | - | - | - |
| *A. sporophleum* | MW208945 | MW221928 | MW221921 |
| *A. subglobosa* | KR069112 | - | - |
| *A. subroseum* | KY494740 | KY705208 | KY705136 |
| ***A. taeanense*** | **MH498515** | **MH498473** | **MH544662** |
| *A. thailandicum* | KY494714 | KY806200 | KY705113 |
| *A. trachycarpum* | MK301099 | MK303395 | MK303397 |
| *A. urticae* | AB220245 | AB220292 | - |
| *A. ushuvaiense* | - | - | - |
| *A. vietnamense* | KX986096 | KY019466 | - |
| *A. xenocordella* | KF144925 | KF145013 | KF145055 |
| *A. yunnanum* | NG_057104 | - | - |
